# Improving emotion recognition in schizophrenia with “VOICES”: An on-line prosodic self-training

**DOI:** 10.1371/journal.pone.0210816

**Published:** 2019-01-25

**Authors:** María Lado-Codesido, Cristina Méndez Pérez, Raimundo Mateos, José Manuel Olivares, Alejandro García Caballero

**Affiliations:** 1 University of Santiago de Compostela, Santiago de Compostela, Spain, Donostia University Hospital, San Sebastián, Spain; 2 University Hospital Complex of Vigo, Vigo, Spain; 3 Department of Psychiatry, School of Medicine, University of Santiago de Compostela, Santiago de Compostela, Spain; 4 Department of Psychiatry, Biomedical Institute of Galicia Sur, Biomedical Research Center in Mental Health Network (CIBERSAM), University Hospital Complex of Vigo, Pontevedra, Spain; 5 Department of Psychiatry, Biomedical Institute of Galicia Sur, Biomedical Research Center in Mental Health Network (CIBERSAM), University Hospital Complex of Ourense, Ourense, Spain; 6 Department of Psychiatry, School of Medicine, University of Santiago de Compostela, Santiago de Compostela, Spain; TNO, NETHERLANDS

## Abstract

**Introduction:**

Emotion recognition (ER) deficits have been extensively demonstrated in schizophrenia. These deficiencies are not only restricted to facial emotion recognition but also include emotional prosody (tone of the voice) recognition deficits. They have been significantly associated with symptom severity and poor social functioning. The aim of this study was to test the efficacy, in real clinical conditions, of an online self-training prosodic game included in the Social Cognition rehabilitation program e-Motional Training.

**Method:**

A randomized, single-blind multicenter clinical trial was conducted with 50 outpatients with schizophrenia or schizoaffective disorder. The control group was treated with Treatment-as-usual (TAU), based on drug therapy, case management and individual and group psychotherapy (not focused on Social Cognition). The intervention group was treated with TAU plus the employment of *Voices*, an online self-training program devised for prosodic rehabilitation.

**Statistical analysis:**

Linear regression was used to evaluate the effectiveness of the intervention in emotion recognition measured with the Reading the Mind in the Voice–Spanish Version (RMV-SV).

**Results:**

There were statistically significant differences between the intervention and control group measured with RMV-SV (β = 3,6[IC 95%], p<0.001), with a response variable in RMV post R^2^ = 0,617.

**Discussion:**

*Voices*, a prosodic rehabilitation game included in e-Motional Training, seems to be a promising tool for improving emotional voice recognition deficits in schizophrenia, filling the need for such interventions.

## 1. Introduction

Social cognition (SC) is defined as the set of mental operations that underlie social interactions and includes the processes involved in the perception, interpretation and generation of responses when faced with the intentions, dispositions and behaviors of others [1, 2]. As with other mental disorders, SC disorders occurring in schizophrenia entail a reduced ability to adapt to social situations, considering the individuals involved and the context in which these situations occur. This reduced ability entails poor functioning and deficient social integration [3] and is a determinant in the quality of life of individuals with schizophrenia [4].

SC includes the following subdomains: theory of mind, social perception, social knowledge or social scheme, attributional bias and emotion recognition [5]. Although all of these subdomains are affected in schizophrenia [5, 6], emotion recognition is considered a potential key factor of social dysfunction in schizophrenia [7]. Research in this field has been largely based on studies on the recognition of the facial expressions of emotions, relegating to second place other perceptive channels, such as the auditory channel [8]. However, auditory recognition could present a greater disorder in schizophrenia than its visual analog [9–13]. According to some authors, however, this disorder depends on the type of emotion expressed [14].

Within auditory perception, we can differentiate the transmission of verbal communication, referring to the content of speech (what is being said), from nonverbal communication or prosody (how it is being said), which includes nonlexical signals within the spoken language, such as accentuation and tone. Prosody fulfills a key function within the organization and interpretation of speech and is of considerable importance for perceiving the emotional state and intentions of others [15].

Prosodic disorders in schizophrenia have been the subject of various studies [12, 16–24] and occur in early stages of the disease and are a stable deficit [25–28]. Although these disorders are greater in patients with chronic disorders[19, 29, 30], they are found in groups with a high risk of psychosis [31] and in first-degree relatives of patients with schizophrenia [32]. The underlying mechanisms and the correlation with neural substrates of these deficits have also been reported [33–35]. These disorders occur at both the expressive [36] and receptive prosody level [11, 12, 18, 37, 38], with a greater difficulty in perceiving emotions with negative content (sadness, fear, anger) [26, 39, 40].

The study of these deficiencies and the search for strategies to improve them are important because SC appears to have a greater repercussion on social functionality than neurocognition by itself [41–43]. Moreover, auditory cognitive training can generate and maintain generalized cognitive improvements [44, 45], causing changes at the cortical level [46, 47].

Even though there is a growing interest in prosodic training, the newly developed rehabilitation programs such as Social Cognition and Interaction Training [48] or Cognitive Enhancement Therapy [49, 50] that included prosodic rehabilitation modules, did not specifically assess the efficacy of the prosodic intervention.

More recently, a description of the Socialville program was published [51, 52], which includes exercises for identifying vocal emotions, using phrases with neutral content expressed with a certain emotional tone. After the phrase has been spoken, between 2 and 5 emotional labels are presented: the objective emotion and distractors, where the patient selects the correct emotion. The game works with basic emotions (neutral, happiness, sadness, anger and fear). The difficulty increases based on the user’s success.

We also found a number of studies that have developed an auditory training program [44, 53] based on the discrimination of stimuli at different frequencies, varying the speed of emission and the interstimulus interval in various exercises ranging from lesser to greater complexity, until a maximum is reached that requires memorizing conversations with a considerable number of details. However, these works [44, 53] are focused on cognitive remediation and not in emotion recognition or prosodic rehabilitation. The study by Sacks et al. [44] showed improvements in SC measures, but these can be attributed to a computerized social cognition module added to the original auditory training.

For the development of these prosodic tools, the use of computerized methods is considered relevant, due to their ease and flexibility of use, reduced costs and easy accessibility [54]. Also relevant are the use of repetitive practices with a high level of dosing, a focus on sensory processing and carefully restricted and individual adapted learning trials [46].

In this article, we present a simple, blind, randomized clinical trial conducted with patients with schizophrenia, using the *Voices* program. The study goals were 1) to assess the efficacy of the program when improving prosodic recognition measured with the RMV–SV and 2) to assess the ease of use and user satisfaction with the program.

## 2. Material and methods

**Fig 1:**
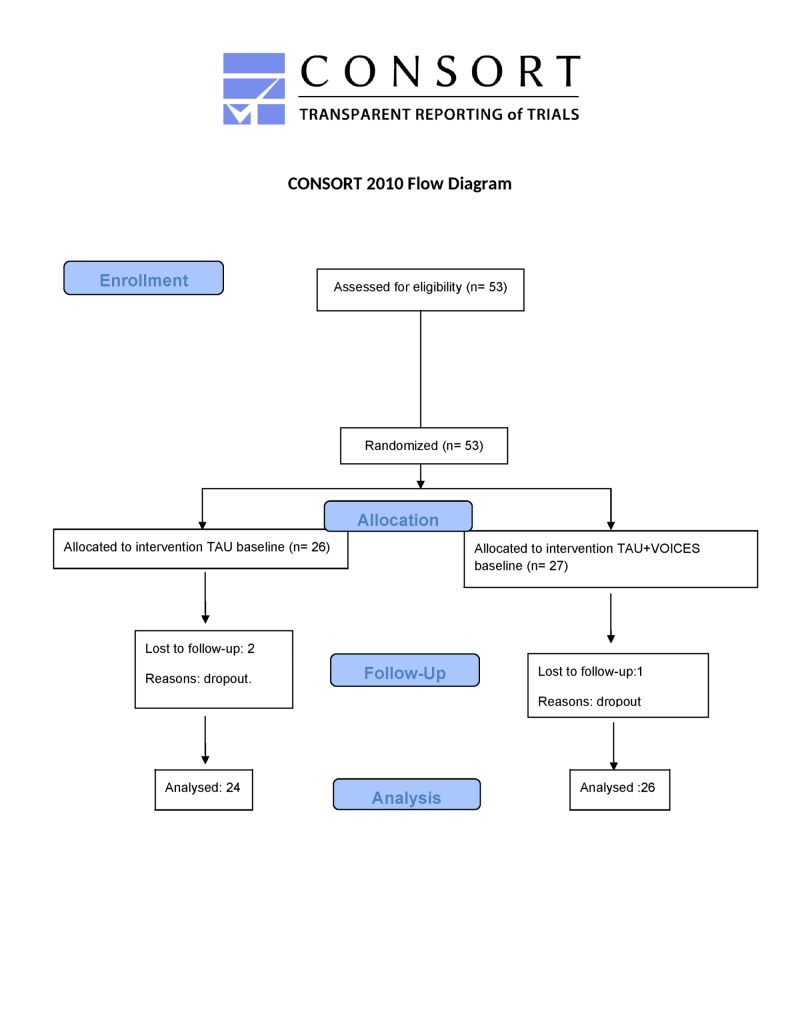
CONSORT flowchart.

We conducted a randomized single-blind multicenter clinical trial that selected 53 patients with schizophrenia or schizoaffective disorder. Fifty-three patients were approached and none of them declined to participate in this study. The patients were recruited from the following 5 centers: University Hospital of Ourense, University Hospital Complex of Ferrol, University Hospital Complex of Vigo, the Ceboliño residence (Ourense) and the Professional Association of Patients with Mental Illnesses of Carballo (A Coruña). After recruitment, the sample was randomized in each center into two balanced groups (Fig 1). Three of the recruited patients were lost to follow-up, 2 in the control group and 1 in the intervention group (control n = 24, intervention n = 26). Therefore, the final sample was composed by 50 patients.

### 2.1 Inclusion and exclusion criteria

We included patients who voluntarily agreed to participate in the study, were 18–60 years of age, had a diagnosis of schizophrenia or schizoaffective disorder (Diagnostic and Statistical Manual of Mental Disorders, 5th Edition), were clinically stable (followed-up by the Department of Psychiatry and undergoing drug treatment), presented an IQ within normal ranges, had no comorbidity with other psychiatric, neurological or severe auditive diseases and who had no current substance abuse (except nicotine). We excluded patients with legal disability. All participants were clinically stable, during the informed consent process, the researchers confirmed that they understood the voluntariness of their participation and the randomization strategy. Written informed consent was therefore obtained by researchers not pertaining to the clinical staff in order to minimize social desirability biases. The recruitment began in January 2017 and ended in April 2017.

### 2.2 Initial database characterization

The mean age was 40.9 (SD = 12.1 years). Fifty-two percent of the participants were men, and 48% were women. Ninety-percent of the participants had been diagnosed with schizophrenia, while 10% had been diagnosed with schizoaffective disorder. Seventy-six percent of the participants presented no associated comorbidity.

#### 2.2.1 Intervention description with *Voices*

The control and intervention groups underwent TAU including drug therapy, case management and individual and group psychotherapy not focused on social cognitive rehabilitation. The control and intervention groups were distributed according to a computer-generated randomization list. The allocation sequence was randomly assigned and concealed from the researcher.

The intervention group underwent 8 sessions of the *Voices* program, divided into 2 weekly sessions lasting approximately 30 minutes for a month. The participants attended their reference center to undergo the training, generally on nonconsecutive weekdays. A common data collection protocol was established for all centers involved in the study. The training was conducted with a personal computer and headphones for every participant, in a peaceful, quiet room, with the support of trained personnel to show the participant how to use the application.

**Fig 2:**
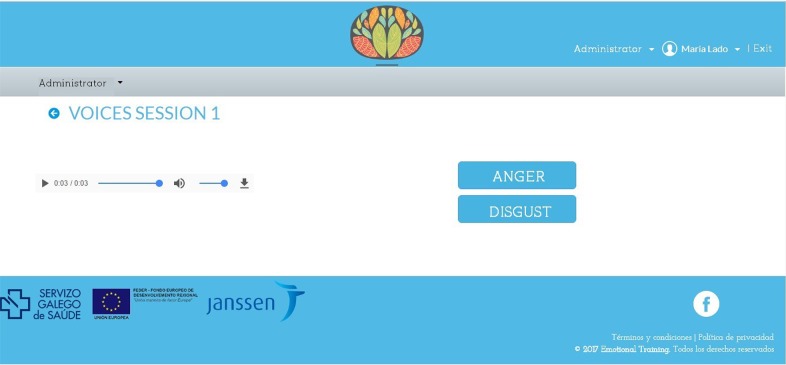
Sessions for *Voices*. Reprinted from www.e-motionaltraining.com under a CC BY license, with permission from Fundación Biomédica Galicia Sur, original copyright 2018.

**Fig 3:**
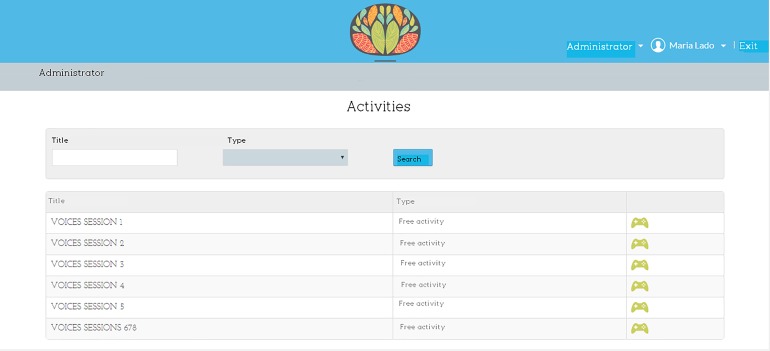
*Voices* program. Reprinted from www.e-motionaltraining.com under a CC BY license, with permission from Fundación Biomédica Galicia Sur, original copyright 2018.

*Voices* (Fig 2) is a computer program consisting of 8 sessions. At the start of each session, the program automatically plays a phrase and after hearing it the participant has to choose the response that corresponds with the emotion conveyed (Fig 3). After that, the program reports whether the choice was correct or incorrect and automatically presents the next fragment for listening. Once the fragments for each session have been completed, the score is displayed. The sessions increase in difficulty gradually (Table 1). The fragments can be listened to as many times as desired. There is no time limit to answer, although once the user has answered, they cannot go backwards.

**Table 1:** Characteristics of the training sessions.

Training session	Number of fragments	Number of response options	Observations
**Session 1**	20	2	Simple dichotomous emotions (happiness/sadness, anger/fear, disgust/neutral)
**Session 2**	20	2 and 3	Simple and complex emotions
**Session 3**	20	3	Complex emotions
**Session 4**	20	3 and 4	Complex emotions
**Session 5**	20	4	Complex emotions
**Sessions 6, 7, 8**	45	2, 3 and 4	Simple and complex emotions *Sessions 6, 7 and 8 use the same type of game with randomized sample options.

Supervision of intervention group was conducted by the center’s staff as a routine activity, and researchers were blind to the assignment.

In order to build the program up, we selected 100 simple and ambiguous phrases (e.g. “I have brought what you asked me”, “It was there, behind the cupboard”) that were recorded by professional actors according to the requested emotion. These audio recordings and the response options were tested with 20 independent examiners. The phrases that had an inter-examiner concordance greater than 70% were selected for the new version, which was then applied to a sample of 164 healthy students recruited from the Faculty of Medicine of the University of Santiago of Compostela, Spain. Subsequently, the phrases that obtained a concordance equal or greater than 80% were selected. These phrases (N = 63) were therefore used to configure the *Voices* program, included in the rehabilitation platform www.e-motionaltraining.com

### 2.3 Evaluation tools

Several tools were employed to assess the benefits of performing prosodic training for patients with schizophrenia and employing *Voices* for this task.

#### 2.3.1 Patients evaluation

All patients were assessed before the intervention, using the instruments described below:

- Customized datasheet designed by the authors for recording demographic and clinical data, including sex, age, occupation, last academic year completed, marital status, present cohabitation, diagnosis and potential associated diagnoses, treatments and equivalence of antipsychotic treatment to chlorpromazine. The initial database characterization was obtained from this demographic data. This information was collected during a conventional clinical interview with the patient and from their electronic medical history.
- *Reading Mind in the Voices–Spanish Version*, *RMV-SV*. Adaptation of the Reading Mind in the Voices–Test Revised (RMV-TR) tool to Spanish [55], which includes 33 segments translated and adapted from English and recorded by professional actors, with 4 response options, with simple and complex emotions. This test will be applied before and after the intervention.
- *Positive and Negative Symptom Scale (PANSS)* [56]: assesses the positive and negative symptom severity.
- *Kauffman Brief Intelligence Test (K-BIT)* [57]: includes the measure of verbal and nonverbal intelligence in adults.

#### 2.3.2 Usability test

When developing software tools, their usability must be considered, including efficiency, ease-of-use and effectiveness. Many user experience questionnaires can be found in the literature, evaluating items such as user satisfaction, usefulness and attractiveness [58, 59].

To evaluate the *Voices* tool, a user experience questionnaire was employed. Thus, participants rated several questions on a Likert-type scale (ranging from 1 = total disagreement, to 5 = total agreement), such as frequency of Internet and computer use (0 = never/almost never, 5 = every day) and ease of connection, understanding of the program interface, autonomy, entertainment, usefulness, global perceived improvement, perceived improvement in social relationships, improvement at work, suitability of the duration and improved self-esteem after applying the program.

#### 2.3.3 Calculating the equivalence of antipsychotic dosages

To convert the various dosages of antipsychotic agents to chlorpromazine, we reviewed the datasheets of the various drugs and conducted a literature review on antipsychotic dosage equivalence [60–63]. The main limitation of this task was the lack of consensus among the various authors for establishing these equivalences, specifically among the more recently marketed antipsychotic agents and long-acting antipsychotic agents [64], where there is a marked lack of information.

### 2.4 Sample size

Given that the literature has no prosodic rehabilitation instruments or training programs similar to the one we proposed, we used the results of the validation of the English version of the RMV-TR test to calculate the sample size [55]. In our case, we hypothesized that the expected difference of the means between the intervention group and the control group in the post-test would be analogous to the difference of the means between the control group without disease and the autism group of the Golan study [55] (Tables 2 and 3). We based this hypothesis on previous facial emotion recognition studies in which the treated group progressed to behaving like the healthy control group after the intervention.

**Table 2:** Sample size Calculation I.

Expected standard deviation	
Population A	3210
Population B	2410
Expected difference of means	3690
Ratio between samples (B/A)	1000
Confidence level	95.0%

**Table 3:** Sample size Calculation II.

Power, %	Sample size	
	Population A	Population B
95.0	16	16
96.0	17	17
97.0	18	18
98.0	20	20
99.0	22	22

### 2.5 Ethical aspects

This study has been conducted in accordance with national and European legislation on clinical research, following international ethical recommendations, the Declaration of Helsinki and the Council of Europe regarding the Convention on Human Rights and Biomedicine. The study complied at all times with the requirements established in the Spanish legislation in the field of biomedical research, personal data protection and bioethics. This study was approved by the local ethics committee (Clinical Research Ethics Committee of Galicia, Registration code: 2016/548) in January 2017 and registered in an international RCT database (BioMed Center: ISRCTN10712315). The clinical trial registry number is https://doi.org/10.1186/ISRCTN10712315. Since the requirement of international registration was unknown for authors, this task was slightly delayed. The authors confirm that all ongoing and related trials for this intervention are registered. Patients' ability to understand the voluntariness of the study was assessed by researchers employing clinical interview. The treating psychiatrists' opinion, based on the patients' clinical history, was also considered.

### 2.6 Statistical analysis

To validate the results from each of the previous studies, we performed statistical analyses to verify whether significant differences were present between the study groups, employing SPSS 22.0 [65] and Epidat 4.1 [66]. For this task, different tests were applied, and p-values were calculated. A general statistical condition for two sets to be significantly different with respect to a given variable is that the p-value [67] is less than 0.05, which means that the null hypothesis (no differences among datasets) can be rejected. This constraint was considered for the calculation of statistical differences for both types of subsets.

The following analyses were performed:

- Descriptive analysis: The qualitative variables are expressed as frequencies and percentages. The continuous variables are expressed as means (standard deviation) or medians (range). To determine the normality of the variables, we performed the Kolmogorov-Smirnov test or the Shapiro-Wilk test.
- Parametric/nonparametric test: subgroup analysis, to determine the homogeneity or heterogeneity between the study groups (chi-squared, Fisher’s exact test, Student's t-test for independent samples and the Mann-Whitney U test).
- Linear regression: to compare the groups pre-post intervention using the RMV-SV test as the dependent variable.

## 3. Results

A total of 53 participants were selected and assigned to control group (TAU) or to the intervention group (*Voices+*TAU). Three of them did not finish the clinical trial (two of control group and one of intervention group), therefore statistical analysis has been performed over a sample of 50.

As stated earlier, the main goal of this research study was to assess the benefits of *Voices* in two different ways. The first was to verify whether the clinical use of the program could help people with a mental disorder improve their social abilities. The second was to measure the usability of *Voices*, in terms of user-friendliness, efficiency, and ease-of-use. The results are presented in the next subsections.

### 3.1. Clinical results

The various data analyses yielded varying results, which are presented in the following paragraphs.

**Fig 4:**
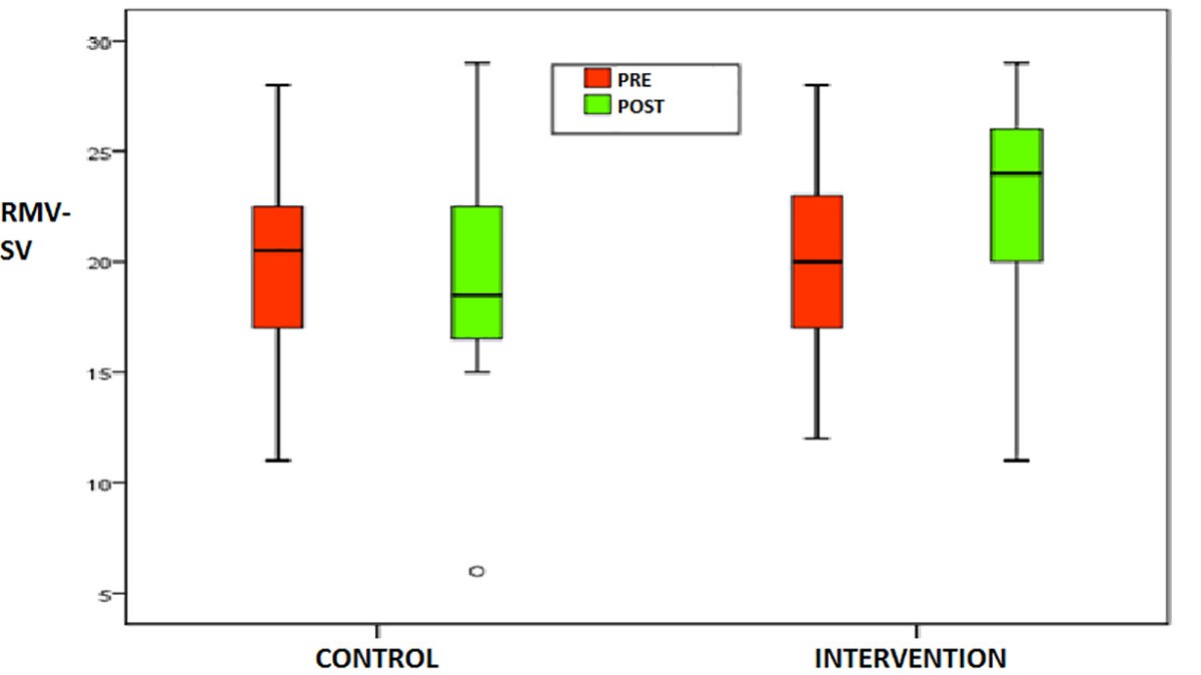
RMV-SV results for both control and intervention group. The left part of the figure represents RMV-SV scores of the control group and the right part represents the RMV-SV scores of the intervention group. The red and green boxes contain the fifty per cent of the results of the RMV-SV scores pre and post-intervention in the different groups. Whiskers indicate the extreme scores of the RMV-SV test.

- Descriptive analysis: in terms of the overall sample (n = 50 patients), 90% of the patients presented a diagnosis of schizophrenia, and 76% presented no associated diagnoses.
  All patients were undergoing therapy with neuroleptics, with a chlorpromazine equivalence of 1008.30, SD = 652.75 mg. At the start of the study, 44% of the patients were undergoing therapy with benzodiazepines, 52% with antidepressants and 18% with mood stabilizers, and 72% were undergoing psychotherapy (Table 4).
- Parametric/nonparametric test: subgroups analysis. Overall statistically significant differences were not found among the intervention and control groups from all centers. No differences were found in the demographic and clinical variables among the groups (Table 5).
- Linear regression: To demonstrate the presence of differences in prosodic variables after treatment, we compared pre-post changes between the two groups, adjusting by initial values of RMV-SV. These results indicate that there were statistically significant differences in score changes between the intervention and control group in RMV-SV (β = 3,6[IC 95%], p<0.001) with a response variable in RMV post R^2^; = 0,617 (Table 6), (Fig 4).

**Table 4:** Sample description.

Demographic characteristic	Percentage
Sex	Male: 52%
	Female: 48%
Age, years (SD)	40.9 (12.1)
Occupation	Active: 6%
	Inactive: 88%
	Students: 4%
	Marginal activities: 2%
Education level	Without primary studies: 8%
	Primary studies: 22%
	Secondary studies: 62%
	University studies: 4%
	Unknown: 4%
Marital status	Single: 84%
	Married: 6%
	Widowed: 2%
	Separated: 8%
Familiar coexistence	Alone: 8%
	With parents: 54%
	As a couple: 8%
	Siblings: 2%
	Others: 28%
**Clinical characteristics**	
Diagnosis	Schizophrenia: 90%
	Schizoaffective disorder: 10%
Associated Diagnosis	No other diagnosis: 76%
	Substance-related and addictive disorder (in the past): 10%
	Mood disorders: 2%
	Somatic symptom and related disorders: 2%
	Personality disorders: 8%
	Neurodevelopmental disorders: 2%
Equivalence to chlorpromazine, mg (SD)	1008.30 (652.75)
Oral treatment, mg.	632.61 (548.17)
Injectable treatment, mg.	972.00 (476.50)
Benzodiazepines	Yes: 44%
	No: 56%
Antidepressants	Yes: 52%
	No: 48%
Mood stabilizers	Yes: 18%
	No: 82%

**Table 5:** Demographic and clinical characteristics by subgroups. means and standard deviations are presented for continuous variables.

VARIABLE		GROUP		p-value
		INTERVENTION (n = 26)	CONTROL (n = 24)	
**Sex**	Male	13	13	0.768
	Female	13	11	
**Age**		40.7 (12.22)	41.2 (12.14)	0.969
**Marital status**	Single	23	19	0.451
	Married	2	1	
	Widowed	0	1	
	Separated	1	3	
**Current cohabitation**	Alone	2	2	0.717
	As a couple	2	2	
	With family	15	13	
	Others	5	9	
**Occupation**	Active	1	2	0.341
	Inactive	23	21	
	Student	2	0	
	Marginal Activities	0	1	
**Education level**	No studies	1	3	0.362
	Primary	8	3	
	Secondary	16	15	
	University	0	2	
	Unknown	1	1	
**Diagnosis**	Schizophrenia	24	21	0.461
	Schizoaffective disorder	2	3	
**Drug treatment**	Antipsychotics	26	24	
	Benzodiazepines	11	11	0.802
	Mood stabilizers	3	6	0.193
	Antidepressants	15	11	0.402
**CPZ equivalence**		921.7	1102	0.334
		(578.7)	(725.3)	
**PANSS**	PANSS-P	11.8(4.8)	14.0(6.8)	0.194
	PANSS-N	22.6(7.8)	20.9 (8.6)	0.485
	PANSS-C	-11.0(6.8)	-7.0(9.0)	0.084
	PANSS-T	52.3(18.2)	56.7(19.3)	0.411
**K-BIT**		98.8(31.6)	96.9 (33.5)	0.838
**RMV-SV**		20.(4.1)	20.1(4.2)	0.944

Abbreviations: CPZ, chlorpromazine; K-BIT, Kauffman Brief Intelligence Test; PANSS, Positive and Negative Syndrome Scale; RMV-SV, Reading the Mind in the Voice—Spanish version; in brackets, SD.

**Table 6:** Linear regression analysis.

RMV-SV	Control		Intervention		β [IC 95%]	p-value
	Pre	post	pre	Post		
Mean	20.08	19.25	20.00	22.92	3,6 [1,8–6,4]	<0,001
SD	4.23	4.93	4.08	4.43		
Median	20.50	18.50	20.00	24.00		
Min.	11.0	6.0	12.0	11.0		
Max.	28.0	29.0	28.0	29.0		

Abbreviations: β: Regression coefficient for the study group.

Results for the RMV-SV values are provided in Table 7. We can see that there were not significant differences pretest between groups, whereas after training a significant improvement was shown in the intervention group (p = 0.009).

**Table 7:** RMV-SV values before and after the *voices* rehabilitation.

GROUP		RMV-SV pre	RMV-SV post
Control	Mean	20.08	19.25
	Standard deviation	4.23	4.93
	Median	20.50	18.50
	Min.	11.0	6.0
	Max.	28.0	29.0
Intervention	Mean	20.00	22.92
	Standard deviation	4.08	4.43
	Median	20.00	24.00
	Min.	12.0	11.0
	Max.	28.0	29.0
p-value		0.944	0.009

Abbreviations: RMV-SV, Reading the Mind in the Voice—Spanish version.

### 3.2 Users’ experience

Information on the use of the Internet and computers was collected. Sixty percent of the sample had used a computer, and 48% used the Internet almost every day. Forty-nine of the 50 patients completed the training sessions.

**Fig 5:**
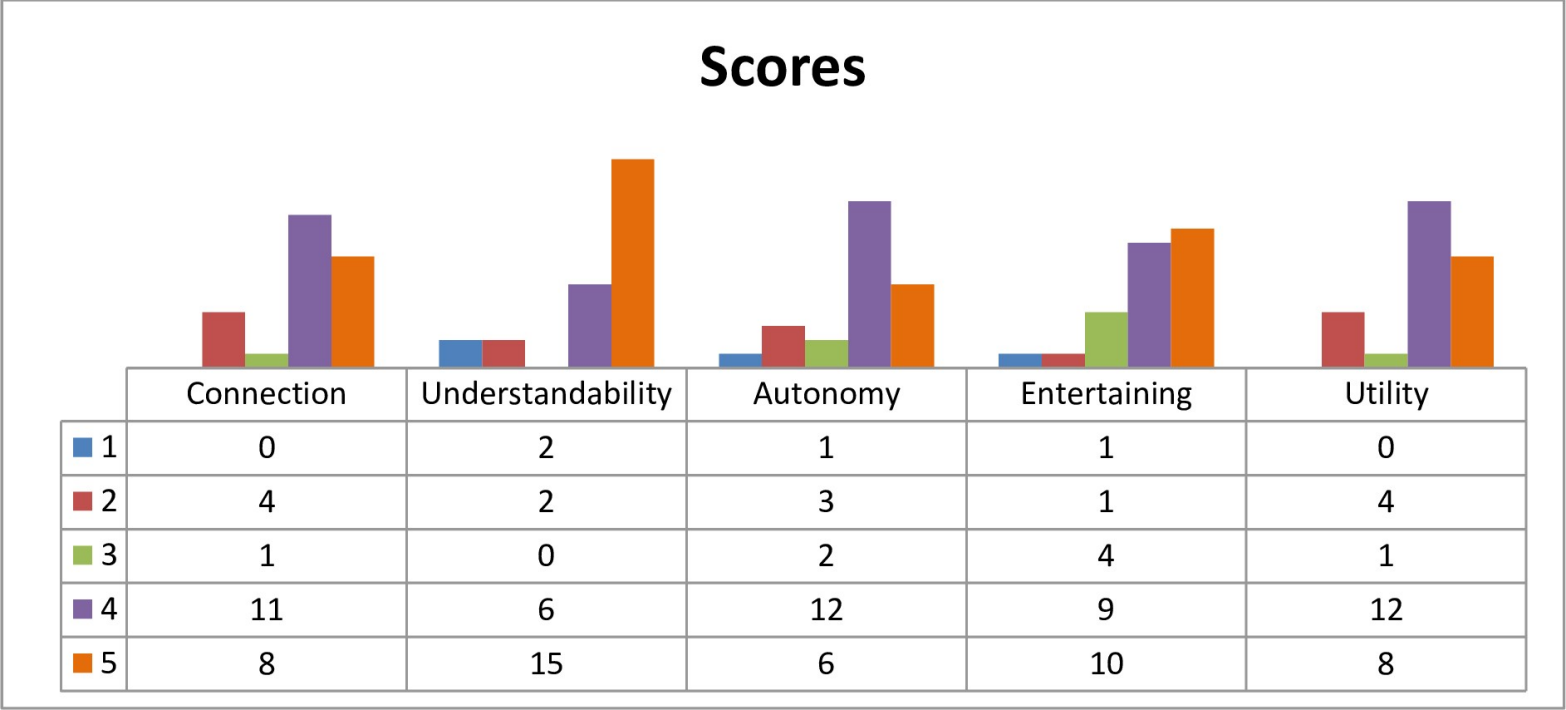
Users’ evaluation of *voices*.

In terms of the use of *Voices*, Fig 5 shows the users’ scores for all items evaluated.

As can be observed, 80% of the patients of the intervention group scored *Voices* with more than 4 points in the easy intelligibility and entertainment items. The item with the lowest score was the program duration, with a mean score of 2.72 out of 5 points.

## 4. Discussion

The results of this study suggest that the use of the *Voices* program improves the prosodic test scores of patients with schizophrenia or schizoaffective disorder compared with patients who are undergoing conventional rehabilitation programs. The use of this type of program could represent an improvement in the level of auditory emotion recognition and could therefore be an advance in the patients’ global psychosocial function.

The patients of the intervention group scored this tool as useful, entertaining and easy to understand. Most of the patients were familiar with the use of the Internet and computers (60% used computers frequently or daily, 48% used the Internet almost daily). This familiarity with information and communication technology might have facilitated good adherence to the program.

This sample consisted of relatively young patients (mean age, 40.9 years). Given that prosody worsens with age [19, 29, 30], younger patients could therefore constitute the key age group for starting prosodic recovery. Early rehabilitation is desirable both for the possibility of stopping the deterioration and for the effects that impaired prosodic recognition could have on global social functioning.

The selected sample presented high PANSS-N scores, which is consistent with the presence of greater prosodic deficiencies according to the reviewed literature [68–70].

In terms of sex, we observed in the literature that male patients presented greater prosodic impairment than female patients [9, 70–72]. In our study, there were no statistically significant differences in the subanalysis by sex, as with other reviewed studies [30].

At the diagnostic level, our patient sample included those diagnosed with schizophrenia and schizoaffective disorder, even though these are different diagnostic entities. In the reviewed studies, we detected prosodic disorders in both diagnoses in stable stages of the disease, as well as lasting deficiencies and markers [40, 73]. We therefore established that this impairment could be rehabilitated in both disorders.

Upon reading our results, we observed that the mean chlorpromazine equivalent dose for the overall sample was 1008.30, SD = 652.75 mg. When dividing the results between oral doses and injectable treatment, we observed that the mean injectable dose was larger than the oral dose (972.00, SD = 476.50 mg vs. 632.61 SD = 548.17 mg). This could be due to the lack of consensus we found in the reviewed literature sources for converting the new formulations of antipsychotic agents (monthly, quarterly, etc.) to the conventional equivalences. An in-depth review on this topic is therefore needed. In the reviewed studies, the antipsychotic treatment was not related to the deficiencies in the SC parameters [74].

We only found an online rehabilitation program (Socialville) that includes prosodic rehabilitation, although it is not its main aim [51]. Regarding prosody the program is focused in recognition of complex emotions, the natural target given that main perceptual difficulties arouse in this type of emotions [17, 28, 75]. Socialville study showed no statistically significant differences pre-post intervention in prosodic rehabilitation (p = 0.09). With our instrument, however, we achieved significance (p = 0.009). The results of the study by Nahum et al. [51] could be attributed to the small sample size (n = 17), given that it was a pilot study. New studies are currently being conducted with larger samples [52]. Moreover, the users of Socialville scored the tool as positive for entertainment, ease of use and satisfaction, results analogous to those obtained for the *Voices* program.

In terms of the other reviewed studies on rehabilitation programs for auditory training [44, 48, 50, 53], we found a number of similarities in the program designs, including the following:

- In the program designed by Sacks et al. [44], in which 50 hours of computerized auditory training plus 12 hours of SC training were performed, the positive results were focused on scores obtained through the Mayer-Salovey-Caruso Emotional Intelligence Test, which assesses overall SC. As mentioned earlier, specific measures of prosody were not collected, although an improvement in this variable would be expected, as it would be in other aspects in SC. This type of study could impel the investigation of the relationship between auditory rehabilitation and overall improvement in other aspects of SC and assess the possibility that auditory rehabilitation acts as a mediator between rehabilitation in overall SC and, as hypothesized in other studies, be a predictor of social functionality [22, 76].
- The program by Fisher et al. [53] conducted 50 hours of intensive computerized training, with an increase in demands at the auditory perception level, compared with 50 hours of computer games. The program observed an improvement in other cognitive variables such as verbal learning and working memory after the auditory training [46]. Unfortunately, as with the study by Sacks et al. [44], the Fisher program did not include prosodic variables that would enable us to compare our results with theirs, although this study supports the hypothesis that exercises in auditory perception could lead to an improvement in other more global cognitive aspects and therefore learning-induced changes in neuroplasticity.
- The previous studies employed a notably high number of hours. Our program had a total duration of 4 hours. Based on the score on the satisfaction scale, we can deduce that the users found the sessions brief. According to Biagianti [45], there was a significant difference in auditory processing speed produced by cognitive therapy starting at 20 hours of training, producing a “plateau phase” between 20 and 40 hours of training. This would indicate that those 20 hours of intervention are optimal, which is quite far from the amount of time we dedicated to our intervention. In the studies we found, however, it is unclear what the minimum number of sessions should be to obtain positive results pre-post intervention on SC. A deeper study along this line is therefore necessary.

### 4.1. Limitations

Our study had a number of limitations. The study was conducted with stable patients, with no active substance abuse, treated with psychoactive drugs, with a mean age of approximately 40 years and in an outpatient regimen treated at outpatient centers. We therefore cannot make conclusions regarding acute and younger patients, those with less overall deterioration, those with substance abuse or those untreated at the pharmacological level, profiles in which prosodic disorders are also observed but in which we do not know the impact of the rehabilitation and, specifically, of our program.

There are a number of limitations inherent in the study topic, such as the lack of prosodic rehabilitation studies conducted to date [8, 37], which considerably limits the comparison with other results from preliminary studies or rehabilitation studies on more cognitive aspects than prosody.

As reflected in the discussion, another limitation is not being able to establish the minimum number of sessions at which improvements in the rehabilitation occur.

Despite the subjective assessment by the patients in terms of their social, occupational and self-esteem improvement after the use of *Voices*, the lack of measurement of other cognitive and function variables hinders our ability to have a more concrete view of the improvements in global function that these strategies can promote.

### 4.2 Conclusions and future work

The patients with schizophrenia or schizoaffective disorder treated with *Voices* significantly improved after the intervention in their prosodic recognition ability, measured with RMV-SV.

Eighty percent of the intervention group scored their satisfaction with the program at 4 or more points in the variables of intelligibility, easy connection, entertainment and usefulness.

This study is part of a series of studies dedicated to developing instruments for rehabilitating SC on the www.e-motionaltraining.com platform. These promising results guide us towards developing new training exercises following this model (*Voices 2*), towards completing a global SC treatment model that covers all SC subdomains, including prosody.

## Supporting information

S1 Checklist CONSORT checklist. (DOC)

S1 Protocol Ethics Comittee protocol of clinical trial in English. (PDF)

S2 Protocol Ethics Comittee protocol of clinical trial in Spanish. (PDF)

S1 Dataset Data analysis of clinical trial. (DOCX)

S2 Dataset Data analysis of clinical trial (ANOVA). (DOCX)

## Abbreviations

CPZ: chlorpromazine
ER: Emotion recognition
K-BIT: Kauffman Brief Intelligence Test
PANSS: Positive and Negative Syndrome Scale
RMV-SV: Reading Mind in the Voice–Spanish Version
RMV-TR: sReading Mind in the Voices–Test Revised
SC: Social cognition
SD: Standard Deviation
TAU: Treatment-as-usual
