# Supplementary material for: Improving emotion recognition in schizophrenia with “VOICES”: An on-line prosodic self-training
Source: PLoS One. 2019 Jan 25;14(1):e0210816. doi: 10.1371/journal.pone.0210816 (PMC6347191; doi:10.1371/journal.pone.0210816)
Supplement: S1 Protocol — (PDF) [file pone.0210816.s002.pdf]

Prosodic rehabilitation program in patients with schizophrenia

Research team:

Alejandro Alberto García Caballero, María Lado Codesido, Luis Miguel Martínez Agulleiro, Isidro Rego Campuzano Rafael Fernández Martínez, Cristina Méndez Pérez

INDEX

1.Introduction

2. Objective

3. Methodology

3.1 Framework for action and sample

3.1.1 Calculation of sample size

3.1.2 Recruitment and selection

3.1.3 Criteria for inclusion

3.1.4 Criteria for exclusion

3.2 Description of the intervention

3.3 Psychometric evaluation

3.4 Handling and treatment of data

3.5 Ethical and legal aspects

3.6 Statistical analysis

3.7 Chronogram

4. Team members

5. Economic data

6. Bibliography

1. INTRODUCTION

Schizophrenia is a mental disorder that causes a series of cognitive deficits related to social functioning in those who suffer from the disorder. Social cognition is defined as the combination of mental operations that lie beneath social interactions, including the processes involved in perception, interpretation, and response generation when faced with the social intentions, disposition and conduct of others. Social cognition has an adaptive function: it allows us to adjust our behaviour when faced with social situations, taking into account the people involved and the context in which such situations occur (Ostrom, 1984; Brothers, 1990). Social behavioural problems frequently precede the onset of psychosis, tend to worsen throughout the course of the illness and contribute to an increased number of relapses (Pinkham et al., 2003).

Social cognition has been an object of study over the last two decades, during which time an effort has been made to describe and analyse its most basic elements. According to the literature reviewed, the main components of social cognition are (Ruiz-Ruiz et al., 2006):

- The *Theory of the* Mind (ToM), which consists in the ability to understand and predict the conduct of other people, their knowledge, intentions and beliefs; that is to say, the capacity to infer the state of mind of others, but also to attribute a mental state to oneself (Brüne, 2003, 2005).
- *Social perception*, which is a term that is applied to the capacity to evaluate rules and social roles, as well as to evaluate the social context. This is the type of perception that requires "*reading between the lines*" (Penn et al. 1997).
- *Social framework,* which is closely related to social perception, is defined as the frame of reference that allows the subject to know how to act, what his or her role is and that of other actors in the situation, what the rules of convention are in a determined situation and the reasons for one's involvement in a social situation (Green et al., 2005).
- The *attributional style,* which concerns the explanations or reasons people give

to the causes of their positive or negative results. It has been observed, for example, in the case of patients with persecutory delusion, that they present a greater tendency to attribute their negative results to other individuals than to the situation (Bentall et al. 2001).

- Finally, our object of study: *emotional processing,* which refers to all aspects related to perception of and use of emotions. There is a deficit that is constant throughout the various stages of the illness but is most acute in moments of crisis, making it a determining factor in social functioning (Edwards et al., 2002).

Empirical knowledge of the recognition and processing of emotions has been fundamentally fuelled by studies on the perception of facial affect through mimicry, relegating other modes of perception, such as auditory perception, to a secondary plane. Within auditory perception we find verbal communication, referring to the content of the discourse ("*what*" is said), and nonverbal communication or prosody ("*how*" it is said), which contains non-lexical signals within spoken language, such as accentuation and tone. Prosody plays a key role in the organization and interpretation of speech, in addition to transmitting the emotional charge contained therein.

In previous literature, a number of studies pertaining to prosody in schizophrenia have centred on discerning in which of the neurological substrati the alteration in the recognition of prosody takes place. The study by Jashan et al. used Auditory Evoked Potentials, observing deficits in the amplitude of the register upon producing tonal processing in patients with schizophrenia compared to the control group. Another area studied is the identification of emotions via tones of Modulated Frequency that mimic characteristics of the human voice with reproduced synthetic tones. Individuals with schizophrenia demonstrated a significant reduction in tonal sensitivity (Kantrowitz et al. 2013). Other studies include the interpretation of emotions via musical expression, in which specific emotional patterns are shown to exist, associated with determined acoustic signals independent from vocal or musical expression (Juslin and Laukka, 2003).

At a neuroanatomical level, some studies demonstrate that the alteration in affective prosody in schizophrenia is produced by the dysfunction of the dominant right hemisphere, in comparison with other study groups with the same deficits and acquired brain damage (Ross et al. 2001). Other studies hypothesise the involvement of the auditory cortex, the inferior frontal gyrus or temporal structures such as the insula or amygdala (Wildgruber, 2006; Kantowitz J., 2015).

At a neuropsychological level, instruments have been developed that attempt to measure recognition of emotions by recreating everyday situations. These instruments were initially designed for the Autistic Spectrum Disorder – where the deficit of social empathy is a fundamental of diagnosis. The main instrument, initially developed by Rutherford et al., is called "*Reading Mind in the Voice"* (RMV), in which 40 segments of conversation between characters in different television series are used. The subject chooses between two words that define the possible mental state of the character. There was a prior adaptation carried out (Golan et al., 2006) called "*Reading Mind in Voices – Text Revised*", in which a wider range of answers was added, increasing validity and reliability in measuring the construct compared to the original instrument.

In terms of the development of these instruments in Spanish, we found a moderate rhythm of recent scientific production in the field of social cognition, in the validation of instruments that, in other languages, have been demonstrated to be effective in evaluating this construct. Examples include the *Hinting Task,* created to evaluate the ToM in Schizophrenia (Gil et al. 2015), and the instrument *Movie for Assessment of Social Cognition* (Lahera, 2014). As far as instruments designed for prosody, we have a few, in the process of validation, such as *Reading Mind in the Voices – Spanish Version,* which is the option closest to the objectives set out in this project.

The study of these deficits, and the research focused on designing strategies to improve them, are important because social cognition seems to have greater repercussions in social functionality than neurocognition itself (Bigelow et al., 2006; Brüne 2005a, Green et al., 2008). The next step is to design therapeutic interventions to diminish these deficits. These interventions have focused primarily on rehabilitation of visual perception, on the basis of programs of repetition or learning a particular

strategy (Paquin y cols. 2014). Programs have been designed to work in groups of 6 – 8 people (*Integrated Psychological Therapy for Schizophrenia -IPT-* Roder y cols., 2007). There are also programs that address all aspects of social cognition (*Social Cognition and Interaction Training -SCIT-* Penn y cols., 2005), in which prosody is worked collaterally when patients receive stimuli through various sensory channels — for example, use of a video clip. Although these programs involve prosody, they do not obtain separate results for prosodic rehabilitation (Vázquez-Campos y cols, 2016).

Among the tools of rehabilitation that deal with prosody, one worth highlighting is *Training in Affect Recognition –TAR-,* which gathers measurements of affective prosody recognition over a 6-week training period, with positive post-intervention results in comparison to other neurocognitive programs (Wölwer y cols., 2005). Another recently published study combines 50 hours of computerized training exercises about auditory perception with 12 hours of identification of emotions, social perception or ToM with positive results in various aspects of social cognition (Sacks y cols, 2014).

Apart from the tools employed for facial recognition, which can be considered "universal", prosody requires tools designed and tested in Spanish. Currently these tools are not available to us, which is why we intend to deepen the study of prosody and its rehabilitation, with the goal of progressing toward the social integration of people with schizophrenia.

2. OBJECTIVE

To evaluate the efficiency of a pilot program (called the *Voices* program) dedicated to prosodic training in patients with schizophrenia or schizoaffective disorder to improve their recognition of basic and complex emotions, resulting, secondarily, in the simultaneous improvement of social cognition and social competencies.

3. METHODOLOGY

3.1. Framework for action and sample

A simple, blind, randomized clinical test will be carried out on patients who

suffer from Schizophrenia or Schizoaffective Disorder and have the legal capacity to consent. Participants in the study will be distributed randomly throughout the experimental group (*Voices*) or the control group, which comes from an ordinary psychosocial rehab program (treatment as usual -*TAU*-).

3.1.1 Calculation of sample size:

The sample will be made up of a group of adults diagnosed with Schizophrenia or Schizoaffective Disorder according to DSM-5 criteria. Given that we do not have instruments of prosodic rehabilitation available in the literature, nor training programs similar to the one proposed here, we will use the results obtained in the validated English version of the RMV-TR test (Golan, 2007) to calculate the sample size. In our case, we hypothesise that the average difference expected between the population of the intervention group and the control population in the post-test will be analogous to the average difference between the control group without illness and the group of patients with autism in Golan's study. For this, we use the findings of previous facial emotion recognition studies in which, through intervention, the treated group comes to demonstrate behaviour like that of the normal control group.

Expected standard deviation
Group A: 3,210
Group B: 2,410
Expected average difference: 3,690
Ratio between samples (B/A): 1,000
Confidence level: 95,0%

	Sample size	
Power (%)	Group A	Group B
95,0	16	16
96,0	17	17
97,0	18	18
98,0	20	20
99,0	22	22

3.1.2 Recruitment and selection:

The participants (in both the experimental and control groups) will be recruited in the following centres:

- **Ourense Healthcare Area:** Psychiatric Day Clinic, University Hospital Complex of Ourense.
- **Ferrol Healthcare Area:** Psychiatric Services Day Clinic, Xerencia de Xestión Integrada of Ferrol.
- **Coruña Healthcare Area:** Psychiatric Day Hospital of the Xerencia de Xestión Integrada of A Coruña and Psychosocial Rehabilitation Centre APEM (Asociación de Pro-Enfermos Mentales) of A Coruña.
- **Vigo Healthcare Area:** Psychiatric Day Clinic of the University Hospital Complex of Vigo.

A member of the research team will contact each candidate, inviting him or her to participate in the study and evaluating the individual's suitability. In the case of acceptance, an appointment will be set in which informed consent will be requested and a pre-evaluation carried out. The selected patients will be randomized by an external collaborator, not involved with the study and belonging to the Unit of Research Support of the Ourense University Hospital Complex, which will be in charge of generating the randomized sequence. The criteria for inclusion will be as follows:

3.1.3 Criteria for inclusion:

- The patient has the capacity to consent.
- The patient has given willing consent to participate in the study, after being informed of the objectives of the study.
- The patient has been diagnosed with Schizophrenia or Schizoaffective Disorder according to the DSM-5 criteria.
- The patient is, at the time of the study, enrolled in Psychiatric Services.
- The patient is receiving neuroleptic pharmacological treatment.
- The patient is within the age range of 18 - 60 years old at the time of participation in the therapy program.

3.1.4 Criteria for exclusion:

- The patient does not give willing consent to participate in the study.
- The patient is incapacitated.

- The patient is suffering from a severe comorbid mental disorder or showing a history of severe brain damage or neurological disorder that could act as a confounding variable, or intellectual disability (examples: organic type associated disorder or diagnosis of limited or low intelligence quotient).
- The patient demonstrates hearing problems.
- The patient is currently participating in a program in skills to improve social adjustment.
- The patient practices active substance abuse.

All patients who meet the criteria for inclusion and none of those who meet the criteria for exclusion will participate.

3.2. Description of the intervention

The training program *Voices* was designed by the initial selection of 100 simple phrases of neutral content; 34 of them contain two answer options in which two simple emotions are expressed, 33 of them contain three options with simple and complex emotions and the last 34 contain a selection of answers in which four complex emotions are expressed. These phrases were later recorded by professional actors based on the emotion prompted. A Microsoft Powerpoint® presentation was created to include these audio recordings and the corresponding answer options. Next, the program was tested with 20 independent testers (11 women and 9 men) and 82 phrases have been extracted and deemed valid (with over 70% agreement among testers). The new version was applied to a sample of 164 healthy control subjects, recruited in the faculty of Medicine at the University of Santiago de Compostela (with 101 women and 63 men, aged 19 – 44 years). On this occasion, answer time was limited to 10 seconds per question. The test duration was approximately 14 minutes. On this occasion, 63 phrases were selected, which achieved a majority agreement of 79.9%.

This definitive selection that defines the *Voices* program has been uploaded to a software platform suitable for patient use, in which a personal user account and password are created. In the *Voices* program, the various phrases are shown randomly,

with the goal of selecting the correct answer of the two, three, or four options. In this selection, there is no set time limit to answer, and there exists the possibility to repeat the audio fragment. At the end of the game, points are registered and compared with the score obtained in previous games.

Eight sessions will be carried out, with a biweekly frequency lasting approximately 20-30 min/day during which time the *Voices* program will be applied in its entirety. In each session, the test content will be randomized.

The test is single blind. The evaluator will be blind to the randomization process that will be carried out by a collaborator not associated with the study. Participation in the research will not cause any increment in regular transportation costs.

3.3. Psychometric Evaluation

All of the main participants and informers will undergo a clinical interview in which general socio-demographical data and clinical information (age, sex, profession, marital status, diagnosis, treatment received in equivalent doses of clorpromazine, etc.), collected in ANNEX I. The objective of collecting this basic demographical data, through the application of the rehabilitation sessions, is to discover if any significant statistical tendency exists that could be an object of further studies.

All patients will be evaluated before and after treatment, via the instruments that will be described below (the definitive set of tools may vary or be reduced according to necessity):

- *Reading Mind in the Voices – Spanish Version*. Adaptation of the RMV-TR tool to Spanish, which includes 33 segments translated and adapted from English and recorded by professional actors, with four answer options, featuring simple and complex emotions.
- *Positive and Negative Symptom Scale (PANSS)*: consists of 30 items that evaluate Schizophrenic syndrome from a double perspective; a dimensional perspective that evaluates the severity of the positive syndrome, the negative syndrome, and the general psychopathology of schizophrenic disorder; and another category that classifies the disorder as positive, negative, or mixed.

- *Kauffman Brief Intelligence Test (K-BIT):* This is a psychometric test whose object is to measure verbal and nonverbal intelligence in children, adolescents and adults. This test is only applied at the beginning, given that modifications of verbal and nonverbal intelligence are not expected.

3.4. Handling and Treatment of Data

Treatment, communication and disclosure of data will be made in accordance with Organic Law 15/1999, regarding protection and regulation of personal data (RD 1720/2007). The data will be encoded such that it cannot be directly identified. The connection between the codes and subjects' identities will remain safeguarded by the IP and only the IP will have access.

3.5. Legal and Ethical Aspects

This study has been designed respecting the rules of good clinical practice and the ethical principles for medical research from the World Medical Association that are reflected in the Declaration of Helsinki and its subsequent revisions. After the same fashion, the European and state regulations are respected, in which medical investigations receive special mention in Organic Law 15/1999 of the 13th of December regarding the protection of personal data. All participating patients will be adequately informed about the purpose of the study and will be asked to sign an informed consent form. The study will be evaluated by the Autonomous Committee for Ethical Investigation (*Comité Autonómico de Ética de la Investigación* or CAEI) of Galicia.

The data will be collected in a consecutive manner in written and electronic form. For this, a database will be used in which participants' information will be encrypted such that the variables collected will not be identified with the participating individuals. The questionnaires and recorded data from each participant will be identified only with the reference number or code. In no case will personal data be published nor revealed to individuals outside of the research or the Committee for Ethical Clinical Investigation.

This study complies with the current Intellectual Property regulations, from the Royal Legislative Decree 1/1996 of the 12th of April.

3.6 Statistical Analysis

A descriptive analysis will be created based on the data. The qualitative variables will be presented with their absolute frequency and percentage. The quantitative Gaussian variables will be presented with their mean and standard deviation (SD), and the non-Gaussian variables will be presented as a median [minimum- maximum]. For the comparison of the two qualitative variables, the McNemar and Chi-squared tests will be used; for the comparison of the quantitative variables, due to the small sample size, tests with U parametrics of Mann-Whitney, Kruskal-Wallis, Wilcoxon and Friedman will be used. Later, a regression analysis will be carried out, using Mixed Models (because although the sample size is very small, mixed models permit data analysis, correcting this problem with multiple measurements per individual). The risk α, accepted for all contrasts of hypothesis, will be 0.05. The data will be analysed with the statistical software package SPSS 15.0 and the Free Software R.

3.7 Chronogram

**Activity**	**Period**				
	October 2016	December 2016	January-April 2017	May 2017	June 2017
Design of protocol	X				
Authorization		X			
Data collection			X		
Statistical analysis and interpretation				X	
Publication					X

4. Team Members

Roles and activities:

- **Alejandro Alberto García Caballero (Developer)**
  - Design and general coordination of the study.
  - Supervision of scientific production.

    - Selection of participants in Ourense Area.
- **María Lado Codesido**
    - Selection of participants in Coruña Area.
    - Review of scientific literature.
    - Writing of scientific article.
- **Luis Miguel Martínez Agulleiro**
    - Selection of participants in Ferrol Area.
- **Isidro Rego Campuzano**
    - Selection of participants in Ourense Area.
- **Rafael Fernández Martínez**
    - Selection of participants in Vigo Area.
- **Cristina Méndez Pérez**
    - Selection of participants in Vigo Area.

5. ECONOMIC DATA

The researchers declare that they have no conflicts of interest. The researchers commit to presenting and publishing the results of said study in journals related to the topic of interest. Given that the study is carried out within the public health care system and does not form part of any clinical program with the goal of commercialising of any medicine or healthcare product involved in the research, tax exemption will be requested.

6. BIBLIOGRAPHY

Bentall, R., Corcoran, R., Howard, R., Blackwood, N., Kinderman, P. 2001. "Persecutory delusions: a review and theoretical integration". *Clinical Psychology Review*, 21, 1143-1192.

Birchwood M, Smith J, Cochrane R, Wetton S, Copestake S. Br J Psychiatry. 1990 Dec;157:853-9. Versión española de: Torres A, Olivares JM. 2005. The Social Functioning Scale. The development and validation of a new scale of social adjustment for use in family intervention programmes with schizophrenic patients. Validation of the Spanish version of the Social Functioning Scale. *Actas Esp Psiquiatr.* Jul-Aug;33(4):216-20.

Bigelow, N.O., Paradiso, S., Adolphs, R., Moser, D.J., Arndt, S., Heberlein, A., Andreasen, NC. 2006. Perception of social relevant stimuli in schizophrenia. Schizophrenia Research, 83, 257-267. http://dx.doi.org/10.1016/j.schres.2005.12.856.

Brothers, L. 1990. “The social brain: a project for integrating primate behaviour and neurophysiology in new domain.” *Concepts in Neuroscience*, 1, 27-61.

Brüne, M. 2003. “Theory of mind and the role of IQ in chronic disorganized schizophrenia”. *Schizophrenia Research*, 60. 57-64.

Brüne M., 2005. “Emotion recognition, “theory of mind” and social behaviour in shizophrenia.” *Psychiatry Research*, 133. 135-147.

Edwards, J., Jackson H.J., Pattison, P.E. 2002. “Emotion Recognition via facial expression and affective prosody in schizophrenia: A methodological review.” *Clinical Psychology Review,* 22, 789-832.

Frommann, N., Streit, M., Wölwer, W. 2003. “Remediation of facial affect recognition impairments in patients with schizophrenia: a new training program”. *Psychiatry Research*, 117, 281-284.

Golan, O., Baron-Cohen, S., Hill, J., Rutherford, M.D., “The “Reading the Mind in the Voice” Test Revised: A Study of Complex Emotion Recognition in Adults with and Without Autism Spectrum Conditions”. 2007. *J Autism Dev Disord.* (37)1096-1106.

Green, M.F., Olivier. B., Crawley, J.N., Penn D.L. y Silverstein, S. 2005. “Social Cognition in Schizphrenia: Recomendations from the Measurement and Treatment Research to Improve Cognition in Schizophrenia New Approaches Conference”. *Schizophrenia Bulletin* 31, 882-887.

Green, M.F., Penn, D.L., Bentall R., Carpenter W.T., Gaebel, W., Gur, C.,...Park, S., 2008. Social cognition in shizophrenia: an NIMH workshop on definitions, assessment, and research opportunities. Schizphrenia Bull. 34 (6). 1211-1220. http://dx.doi.org/10.1093/schbul/sdm.145

Ruiz-Ruiz, JC; García-Ferrer, S.; Fuentes-Durá, I. 2006. La relevancia de la cognición social en la esquizofrenia. *Apuntes de Psicología.* Vol.24 (1-3); 135-155.

Rutherford, M.D., Baron-Cohen S., Wheelwright, S., 2002. “Reading the Mind in the Voice: A Study with Normal Adults and Adults with Asperger Syndrome and High Functioning Autism”. Journal of Autism and Developmental Disorders, (32), 3, 189-194.

Sacks S, Fisher M, Garrett C, Alexander P, Holland C, Rose D, Hooker C, Vinogradov S.“Combining computerized social cognitive training with neuroplasticity-based

auditory training in schizophrenia.” Clin Schizophr Relat Psychoses. 2013 Summer;7(2):78-86A.

Vázquez-Campo, M., Maroño, Y., Lahera, G., Mateos, R., García-Caballero, A., 2016. “e-Motional Training: a pilot study on a novel online training program on social cognition for patients with schizophrenia”. *Schizophrenia Research: Cognition*. (4) 10-17.

Wölwer, W., Fromman, N., Halfmann, S., Piaszek, A., Streit, M., y Gaebel, W. 2005. “Remediation of impairments in facial affect recognition in schizophrenia: Efficacy and specificity of a new training program.” *Schizophrenia Research* (80), 295-303.
