# Supplementary material for: Improving emotion recognition in schizophrenia with “VOICES”: An on-line prosodic self-training
Source: PLoS One. 2019 Jan 25;14(1):e0210816. doi: 10.1371/journal.pone.0210816 (PMC6347191; doi:10.1371/journal.pone.0210816)
Supplement: S2 Protocol — (PDF) [file pone.0210816.s003.pdf]

PROGRAMA DE ENTRENAMIENTO DE REHABILITACIÓN PROSODICA EN PACIENTES CON ESQUIZOFRENIA

Equipo Investigador:
Alejandro Alberto García Caballero, María Lado Codesido, Luis Miguel Martínez Agulleiro, Isidro Rego Campuzano Rafael Fernández Martínez, Cristina Méndez Pérez

INDICE

1.Introduccion

2. Objetivo

3. Metodología

3.1 Marco de actuación y muestra

3.1.1 Cálculo del tamaño muestral

3.1.2 Reclutamiento y selección

3.1.3 Criterios de inclusión

3.1.4 Criterios de exclusión

3.2 Descripción de la intervención

3.3 Evaluación psicométrica

3.4 Manejo y tratamiento de datos

3.5 Aspectos éticos y legales

3.6 Análisis estadístico

3.7 Cronograma

4. Miembros del equipo

5. Datos económicos

6. Bibliografía

1. INTRODUCCIÓN

La esquizofrenia es un trastorno mental donde existen una serie de déficits cognitivos que se correlacionan con el funcionamiento social de las personas que la padecen. La llamada cognición social, se define como el conjunto de operaciones mentales que subyacen a las interacciones sociales, y que incluyen los procesos implicados en la percepción, interpretación y generación de respuestas ante las intenciones, disposiciones y conductas de otros (Ostrom, 1984; Brothers, 1990). Tiene una función adaptativa, dado que nos permite flexibilizar nuestra manera de actuar frente a las situaciones sociales, teniendo en cuenta las personas implicadas y el contexto en el que suceden dichas situaciones. Los problemas comportamentales de tipo social, frecuentemente preceden también al desencadenamiento de la psicosis, suelen empeorar en el transcurso de la enfermedad y contribuyen a aumentar el número de recaídas (Pinkham y cols. 2003).

Este aspecto cognitivo ha sido objeto de estudio en las dos últimas décadas, donde se ha tratado de describir y analizar sus elementos más básicos. Según la bibliografía revisada, los principales componentes que se forman parte de la cognición social son (Ruiz-Ruiz y cols., 2006):

- La *Teoría de la Mente* (ToM), consiste en la habilidad para comprender y predecir la conducta de otras personas, sus conocimientos, intenciones y creencias, es decir, la capacidad para inferir cuál es el estado mental de los demás, pero también para atribuirse a sí mismos un estado mental. (Brüne, 2003, 2005).

- La *percepción social,* es un término que se aplica a las capacidades para valorar reglas y roles sociales, así como para valorar el contexto social. Es el tipo de percepción que requiere "*leer entre líneas*" (Penn y cols. 1997).

- El *conocimiento* o *esquema social*, muy ligado a la percepción social, que se define como el marco de referencia que permite al sujeto saber cómo se debe actuar cuál es su papel y el de otros actores en la situación, cuáles son las reglas que por convención se utilizan en una situación determinada y cuáles son las

razones por las cuales se ve implicado en una situación social (Green y cols., 2005).

- El *estilo atribucional*, se ocupa de la forma con la que se valoran las explicaciones o razones que uno da a las causas de sus resultados positivos o negativos. Se ha observado, por ejemplo en el caso de los pacientes con delirios persecutorios, que presentan una mayor tendencia a atribuir sus malos resultados a otros individuos más que a la situación (Bentall y cols. 2001).

- Por último, nuestro objeto de estudio: el *procesamiento emocional*, que hace referencia a todos aquellos aspectos relacionados con percibir y utilizar emociones. Se trata de un déficit estable en los distintos estados de enfermedad, pero es más acusado en los momentos de crisis, convirtiéndose en un factor determinante en el funcionamiento social (Edwards y cols., 2002).

El conocimiento empírico del reconocimiento y procesamiento de emociones se ha nutrido fundamentalmente de estudios sobre la percepción del afecto facial, mediante la mímica, relegando a un segundo plano otros canales perceptivos, como el auditivo. Dentro de la percepción auditiva, encontramos la comunicación verbal, referida al contenido del discurso (el "*qué*" se dice) y la comunicación no verbal o prosodia (el "*cómo*" se dice), donde se engloban las señales no léxicas dentro del idioma hablado, como la acentuación o el tono. La prosodia cumple una función clave dentro de la organización e interpretación del discurso, además de transmitir la carga emocional del mismo.

Revisando la literatura previa, algunos estudios referidos a la prosodia en esquizofrenia se han centrado en discenir cuál es el sustrato neurológico de la alteración en el reconocimiento de la prosodia. En el estudio de Jashan y cols (2013), se han utilizado los Potenciales Evocados Auditivos, observándose déficits en la amplitud del registro a la hora de producirse el procesamiento tonal en los pacientes con esquizofrenia en relación a los controles. Otro ámbito estudiado es la identificación de emociones a través de tonos de Frecuencia Modulada, característicos de la voz humana, reproducibles mediante el uso de tonos sintéticos, donde también se demostró una reducción significativa de la sensibilidad tonal en los casos

(Kantrowitz y cols. 2013). Existen también estudios donde se incluye la expresión musical y la interpretación de emociones a través de la misma, donde se concluye que existen patrones específicos emocionales que se asocian a determinadas señales acústicas de forma independiente a nivel de expresión vocal o musical (Juslin y Laukka 2003).

A nivel neuroanatómico, algunos estudios hablan de que la alteración de la prosodia afectiva en esquizofrenia se produce mediante la disfunción del hemisferio derecho dominante, en comparación con otros grupos de estudio con los mismos déficits y daño cerebral adquirido (Ross y cols. 2001). En otros estudios, se postula la implicación del córtex auditivo, el giro frontal inferior o estructuras temporales como la ínsula o amígdala (Wildgruber, 2006; Kantowitz J., 2015).

A nivel neuropsicológico, se han desarrollado algunos instrumentos que tratan de medir el reconocimiento de emociones, tratando de recrear situaciones cotidianas. Inicialmente se han diseñado para el Trastorno del Espectro Autista -donde el déficit de empatía social supone un pilar diagnóstico. El principal de ellos, desarrollado inicialmente por Rutherford y cols (2002), se llama "*Reading Mind in the Voice*" (RMV), donde se emplearon 40 segmentos de conversación entre personajes de diferentes series de televisión para que el sujeto elija entre dos palabras, que definen el posible estado mental del personaje. Se realizó una adaptación posterior, realizada por Golan y cols (2006), llamada "*Reading Mind in Voices-Test Revised*", donde se añadió un abanico más amplio de respuestas, incrementando la validez y fiabilidad en la medición del constructo en referencia al instrumento original.

En cuanto al desarrollo de estas herramientas en español, encontramos una moderada y reciente producción científica en el campo de la cognición social, en la validación de herramientas que han demostrado eficacia en otros idiomas para evaluar este constructo. Un ejemplo es el *Hinting Task*, desarrollado para valorar la ToM en Esquizofrenia (Gil y cols 2015), o el instrumento *Movie for Assesmente of Social Cognition* (Lahera, 2014). En cuanto a herramientas diseñadas para la prosodia, disponemos de algunas, en proceso de validación, como el *Reading Mind in the Voices-Spanish Version,* que se convierte en la opción más cercana a los objetivos que se

plantea este proyecto.

El estudio de estos déficits y la investigación enfocada al diseño de estrategias para mejorarlos, son importantes porque la cognición social parece tener una mayor repercusión en la funcionalidad social que la neurocognición por sí misma (Bigelow y cols., 2006; Brüne 2005a, Green y cols., 2008). El siguiente paso, por lo tanto, es el diseño de herramientas terapéuticas para disminuir estos déficits, que se han centrado primordialmente en el rehabilitación de la percepción visual, basándose en programas de repetición o bien mediante el aprendizaje de una estrategia determinada (Paquin y cols. 2014). Se han diseñado programas, donde se trabaja en grupos de 6-8 personas (*Terapia Psicológica Integrada para la Esquizofrenia -IPT-* Roder y cols., 2007) o programas que trabajan todos los aspectos de la cognición social (*Entrenamiento en Cognición social e Interacción -SCIT-*, Penn y cols., 2005), donde colateralmente se trabaja la prosodia, dado que los pacientes reciben estímulos por varios canales sensoriales –sin tener resultados de la variable de rehabilitación prosódica por sí mismos- por ejemplo, en el caso de usar un videoclip para trabajar estos aspectos (Vázquez-Campos y cols, 2016).

Dentro de las herramientas rehabilitadoras donde se recoge la prosodia, destacamos el *Entrenamiento en Reconocimiento del Afecto -TAR-*, donde se recoge la medición del reconocimiento de la prosodia afectiva, con resultados positivos post-intervención en comparación con el uso de otros programas neurocognitivos, tras un entrenamiento de seis semanas (Wölwer y cols., 2005). Otro estudio de reciente publicación, se combina 50 horas de ejercicios de entrenamiento computerizado sobre percepción auditiva con 12 horas de identificación de emociones, percepción social o ToM, con resultados positivos en varios aspectos de la cognición social (Sacks y cols, 2014).

A diferencia de las herramientas empleadas para el reconocimiento facial, que se podrían considerar "universales", la prosodia necesita de herramientas diseñadas y validadas en español. En el momento actual no disponemos de estas herramientas, por lo que nos proponemos profundizar en el estudio de la prosodia y su rehabilitación, con el fin de progresar hacia la integración social de las personas con esquizofrenia.

2. OBJETIVO

Evaluar la eficacia de un programa piloto (llamado programa *Voices*) dedicado al entrenamiento prosódico en pacientes con esquizofrenia o trastorno esquizoafectivo, a la hora de mejorar el reconocimiento de emociones básicas y complejas, y con ello mejorar de forma secundaria la cognición social y las competencias sociales.

3. METODOLOGÍA

3.1. Marco de actuación y muestra

Se realizará un ensayo clínico aleatorizado simple ciego en pacientes que padezcan Esquizofrenia o Trastorno Esquizoafectivo y tengan capacidad legal para consentir. Los participantes en el estudio serán distribuidos al azar al grupo experimental (*Voices*) o al grupo control que se encuentre en un programa de rehabilitación psicosocial ordinario (tratamiento habitual –*TAU*-).

3.1.1 Cálculo del tamaño muestral:

La muestra se constituirá por un grupo de adultos diagnosticados de Esquizofrenia o Trastorno Esquizoafectivo según criterios DSM-5. Dado que no disponemos en la literatura de instrumentos de rehabilitación prosódica ni programas de entrenamiento similares al que planteamos, usaremos para el cálculo del tamaño muestral los resultados obtenidos en la validación de la versión inglesa del test RMV-TR (Golan, 2007). En nuestro caso la diferencia de medias esperada entre la población del grupo intervención y la población control en el postest hipotetizamos que será análoga a la diferencia de medias entre el grupo control sin enfermedad y el grupo de pacientes con autismo del estudio de Golan (2007). Para ello nos basamos en estudios previos de reconocimiento de emociones faciales en los cuales tras la intervención el grupo tratado pasa a comportarse como el grupo control normal.

Desviación estándar esperada
Población A: 3,210
Población B: 2,410
Diferencia de medias esperada: 3,690
Razón entre muestras (B/A): 1,000
Nivel de confianza: 95,0%

Tamaño de muestra		
Potencia (%)	Población A	Población B
---------------	--------------	--------------
95,0	16	16
96,0	17	17
97,0	18	18
98,0	20	20
99,0	22	22

3.1.2 Reclutamiento y selección:

Los participantes (tanto casos como controles) serán reclutados en los siguientes centros:

- **Área Sanitaria de Ourense:** Hospital de Día de Psiquiatría, Complejo Hospitalario Universitario de Ourense.
- **Área Sanitaria de Ferrol:** Hospital de Día del Servicio de Psiquiatría, Xerencia de Xestión Integrada de Ferrol.
- **Área Sanitaria de Coruña:** Hospital de Día de Psiquiatría de la Xerencia de Xestión Integrada de A Coruña y Centro de Rehabilitación Psicosocial de APEM (Asociación de Pro-Enfermos Mentales de A Coruña).
- **Área Sanitaria de Vigo:** Hospital de Día de Psiquiatría del Complejo Hospitalario Universitario de Vigo.

Un miembro del equipo investigador contactará con cada candidato invitándole a participar en el estudio y a realizar una evaluación de idoneidad. En caso de aceptación se concertará una cita en la que se solicitará consentimiento informado y se realizará una preevaluación. Los pacientes seleccionados serán aleatorizados, por un colaborador ajeno al estudio y perteneciente a la Unidad de Apoyo a la Investigación del CHUO, que será el encargado de generar la secuencia de aleatorización. Los

criterios de inclusión serán los siguientes:

3.1.3 Criterios de inclusión:

- El paciente tiene capacidad para consentir
- Haber dado su libre consentimiento a participar en el estudio, tras ser informado de los objetivos del mismo.
- Diagnóstico de Esquizofrenia o Trastorno Esquizoafectivo según criterios DSM-5.
- En el momento del estudio encontrarse a seguimiento en Servicio de Psiquiatría.
- Estar a tratamiento farmacológico con neurolépticos.
- Edad comprendida entre 18 y 60 años en el momento de la inclusión en el programa terapéutico.

3.1.4 Criterios de exclusión:

- No haber dado su libre consentimiento a participar en el estudio.
- Incapacidad.
- Presentar un trastorno mental grave comórbido o presentar historia de daño cerebral severo o trastorno neurológico que pueda funcionar como factor de confusión, o discapacidad intelectual (ejemplos: trastorno asociado de tipo orgánico o diagnóstico de coeficiente intelectual límite o inferior).
- Presentar problemas de audición.
- Participación actual en un programa de habilidades destinadas a mejorar el ajuste social.
- Abuso de sustancias tóxicas activo.

Se incluirán todos los pacientes que cumplan todos los criterios de inclusión y ninguno de los de exclusión.

3.2. Descripción de la intervención

El programa de entrenamiento *Voices* se ha diseñado mediante la selección inicial de 100 frases simples de contenido neutro; en 34 de ellas se han seleccionado dos opciones de respuesta donde se expresan dos emociones simples, en 33 de ellas

tres opciones con emociones simples y complejas y en las últimas 34 una selección de respuestas donde se expresan cuatro emociones complejas. Posteriormente han sido grabadas por actores y actrices profesionales en base a la emoción solicitada. Se han incluído los audios y las opciones de respuesta correspondientes en una presentación de Microsoft Powerpoint®. A continuación, se ha testado con 20 examinadores independientes (11 mujeres y 9 varones) y se han extraído 82 frases consideradas válidas (con una concordancia mayor del 70% interexaminador). La nueva versión fue aplicada a una muestra de 164 controles sanos, reclutados de la facultad de Medicina de la Universidad de Santiago de Compostela (con 101 mujeres y 63 varones, y edades comprendidas entre los 19 y 44 años). En este ocasión, se limitó el tiempo de respuesta a 10 segundo por pregunta. La duración del test fue de aproximadamente 14 minutos. En esta ocasión, se seleccionaron 63 frases, que obtuvieron una concordancia mayor del 79.9%.

Esta selección definitiva que conforma el programa *Voices,* se subió a una plataforma informática apta para el uso de los pacientes, donde se obtiene una cuenta de registro y una clave de usuario personal. En el programa *Voices*, se muestran las diferentes frases de forma aleatoria, con la finalidad de ir seleccionando la respuesta correcta de las dos, tres o cuatro opciones. En esta selección, no existe un tiempo de respuesta estipulado y existe la posibilidad de repetir el audio del fragmento. Al final del juego, se registran las puntuaciones y se comparan con las puntuaciones obtenidas en juegos previos.

Se llevarán a cabo ocho sesiones, con una perioricidad bisemanal, con una duración aproximada de 20-30min/día donde se aplicará el programa *Voices* al completo. En cada sesión variará la aleatorización del contenido del test.

El ensayo es simple ciego. El evaluador será ciego al proceso de aleatorización que será llevado a cabo por un colaborador ajeno al estudio. La participación en la investigación no supondrá por tanto un incremento de los costes de transporte habituales.

3.3. Evaluación Psicométrica

A todos los participantes e informadores principales se les realizará una entrevista clínica en la que se recogerán datos sociodemográficos generales e información clínica (edad, sexo, profesión, estado civil, diagnóstico, tratamiento recibido en dosis equivalentes a clorpromazina, etc.), recogido en ANEXO I. El objetivo de la recogida de estos datos demográficos básicos es conocer si existe alguna tendencia estadísticamente significativa de respuesta, tras la aplicación de las sesiones de rehabilitación, que pudiese ser objeto de otros estudios.

Todos los pacientes serán evaluados antes y después del tratamiento, mediante los instrumentos que se describen a continuación (la batería definitiva puede variar o acortarse en función de necesidades):

- *Reading Mind in the Voices – Spanish Version*. Adaptación de la herramienta RMV-TR al español, donde se incluyen 33 segmentos traducidos y adaptados del inglés y grabados por actores y actrices profesionales, con cuatro opciones de respuesta, con emociones simples y complejas.
- *Escala de Síntomas Positivos y Negativos (PANSS)*: consta de 30 ítems que evalúan el síndrome esquizofrénico desde una doble perspectiva; una dimensional que evalúa la gravedad del síndrome positivo, del negativo, y de la psicopatología general del trastorno esquizofrénico; y otra categorial que lo clasifica en positivo, negativo o mixto.
- *Test Breve de Inteligencia de Kauffman (K-BIT):* es una prueba psicométrica que tiene como objetivo medir la inteligencia verbal y no verbal en niños, adolescentes y adultos. Esta prueba sólo se aplicará al inicio, dado que no es esperable que se observen modificaciones en la misma.

3.4. Manejo y Tratamiento de los Datos

El tratamiento, comunicación y cesión de datos se hará según lo dispuesto en la Ley Orgánica 15/1999, de protección de datos de carácter personal y su reglamento (RD 1720/2007). Los datos serán codificados, de manera que no se puedan identificar directamente. La relación entre los códigos y la identidad del sujeto permanecerá custodiada por el IP y sólo él tendrá acceso.

3.5. Aspectos Éticos y Legales

Este estudio ha sido diseñado respetando las normas de buena práctica clínica y los principios éticos para las investigaciones médicas de la asociación médica mundial que quedan reflejadas en la declaración de Helsinki y sus posteriores enmiendas. Del mismo modo se respetan la normativa Europea y estatal en lo que a investigación médica se refiere con especial mención a la ley orgánica 15/1999 de 13 de diciembre de protección de datos de carácter personal. Todos los pacientes incluidos serán informados adecuadamente sobre la finalidad del estudio y se les pedirá que firmen un consentimiento informado. El estudio será evaluado por el CAEI de Galicia.

Los datos serán recogidos de forma consecutiva en soporte escrito e informático. Para ello se utilizará una base de datos en la que los participantes se codificarán de modo que no se identifiquen las variables recogidas con los individuos participantes. Los cuestionarios, y datos recogidos de cada participante se identificarán siempre y sólo con un número de referencia o código. En ningún caso se verán publicados los datos personales ni se revelarán a personas ajenas a la investigación o al Comité Ético de Investigación Clínica.

El estudio cumple la normativa vigente de Propiedad Intelectual, del Real Decreto legislativo 1/1996 del 12 de abril.

3.6 Análisis Estadístico

Se realizará un análisis descriptivo de los datos. Las variables cualitativas se presentarán con su frecuencia absoluta y porcentaje. Las variables cuantitativas gaussianas se presentarán con su media y desviación estándar (DE) y las no gaussianas como mediana [mínimo- máximo]. Para la comparación de dos variables cualitativas se utilizarán las pruebas de McNemar y Chi-cuadrado; para la comparación de las variables cuantitativas, y debido al pequeño tamaño muestral, se utilizarán las pruebas no paramétricas U de Mann-Whitney, Kruskal-Wallis, Wilcoxon y Friedman. Posteriormente se realizará un análisis de regresión utilizando Modelos Mixtos (ya que aunque el tamaño muestral es muy pequeño, los modelos mixtos permiten analizar los datos, subsanando este problema con las múltiples mediciones por individuo). El riesgo α aceptado para todos los contrastes de hipótesis será de 0,05. Los datos serán

analizados con el paquete estadístico SPSS 15.0 y el Software libre R.

3.7 Cronograma

Actividad	Período				
	Octubre 2016	Diciembre 2016	Enero-Abril 2017	Mayo 2017	Junio 2017
Diseño del protocolo	X				
Autorización		X			
Recogida de datos			X		
Análisis e interpretación estadística				X	
Publicación					X

4. MIEMBROS DEL EQUIPO

Funciones y actividades:

- **Alejandro Alberto García Caballero (Promotor)**
  - Diseño y coordinación general del estudio.
  - Supervisión de la producción científica.
  - Selección de participantes del Área de Ourense.
- **María Lado Codesido**
  - Selección de participantes del Área de A Coruña.
  - Revisión de la literatura científica
  - Redacción de artículo científico
- **Luis Miguel Martínez Agulleiro**
  - Selección de participantes del Área de Ferrol.
- **Isidro Rego Campuzano**
  - Selección de participantes del Área de Ourense.
- **Rafael Fernández Martínez**

    - Selección de participantes del Área de Vigo**.**
- **Cristina Méndez Pérez**
    - Selección de participantes del Área de Vigo.

5. DATOS ECONÓMICOS

Los investigadores declaran no tener conflictos de interés. Los investigadores se comprometen a presentar y publicar los resultados de dicho estudio en revistas relacionadas con el tema de interés. Dado que el estudio se realiza dentro del sistema sanitario público y no forma parte de ningún programa clínico que tenga como fin la comercialización de un medicamento o producto sanitario objeto de investigación, se solicitará la exención de tasas.

6. BIBLIOGRAFÍA

Bentall, R., Corcoran, R., Howard, R., Blackwood, N., Kinderman, P. 2001. "Persecutory delusions: a review and theoretical integration". *Clinical Psychology Review*, 21, 1143-1192.

Birchwood M, Smith J, Cochrane R, Wetton S, Copestake S. Br J Psychiatry. 1990 Dec;157:853-9. Versión española de: Torres A, Olivares JM. 2005. The Social Functioning Scale. The development and validation of a new scale of social adjustment for use in family intervention programmes with schizophrenic patients. Validation of the Spanish version of the Social Functioning Scale. *Actas Esp Psiquiatr.* Jul-Aug;33(4):216-20.

Bigelow, N.O., Paradiso, S., Adolphs, R., Moser, D.J., Arndt, S., Heberlein, A., Andreasen, NC. 2006. Perception of social relevant stimuli in schizophrenia. Schizophrenia Research, 83, 257-267. http://dx.doi.org/10.1016/j.schres.2005.12.856.

Brothers, L. 1990. "The social brain: a project for integrating primate behaviour and neurophysiology in new domain." *Concepts in Neuroscience*, 1, 27-61.

Brüne, M. 2003. "Theory of mind and the role of IQ in chronic disorganized schizophrenia". *Schizophrenia Research*, 60. 57-64.

Brüne M., 2005. "Emotion recognition, "theory of mind" and social behaviour in shizophrenia." *Psychiatry Research*, 133. 135-147.

Edwards, J., Jackson H.J., Pattison, P.E. 2002. "Emotion Recognition via facial expression and affective prosody in schizophrenia: A methodological review." *Clinical Psychology Review,* 22, 789-832.

Frommann, N., Streit, M., Wölwer, W. 2003. "Remediation of facial affect recognition impairments in patients with schizophrenia: a new training program". *Psychiatry Research*, 117, 281-284.

Golan, O., Baron-Cohen, S., Hill, J., Rutherford, M.D., "The "Reading the Mind in the Voice" Test Revised: A Study of Complex Emotion Recognition in Adults with and Without Autism Spectrum Conditions". 2007. *J Autism Dev Disord.* (37)1096-1106.

Green, M.F., Olivier. B., Crawley, J.N., Penn D.L. y Silverstein, S. 2005. "Social Cognition in Schizphrenia: Recomendations from the Measurement and Treatment Research to Improve Cognition in Schizophrenia New Approaches Conference". *Schizophrenia Bulletin* 31, 882-887.

Green, M.F., Penn, D.L., Bentall R., Carpenter W.T., Gaebel, W., Gur, C.,...Park, S., 2008. Social cognition in shizophrenia: an NIMH workshop on definitions, assessment, and research opportunities. Schizphrenia Bull. 34 (6). 1211-1220. http://dx.doi.org/10.1093/schbul/sdm.145

Ruiz-Ruiz, JC; García-Ferrer, S.; Fuentes-Durá, I. 2006. La relevancia de la cognición social en la esquizofrenia. *Apuntes de Psicología.* Vol.24 (1-3); 135-155.

Rutherford, M.D., Baron-Cohen S., Wheelwright, S., 2002. "Reading the Mind in the Voice: A Study with Normal Adults and Adults with Asperger Syndrome and High Functioning Autism". Journal of Autism and Developmental Disorders, (32), 3, 189-194.

Sacks S, Fisher M, Garrett C, Alexander P, Holland C, Rose D, Hooker C, Vinogradov S."Combining computerized social cognitive training with neuroplasticity-based auditory training in schizophrenia." Clin Schizophr Relat Psychoses. 2013 Summer;7(2):78-86A.

Vázquez-Campo, M., Maroño, Y., Lahera, G., Mateos, R., García-Caballero, A., 2016. "e-Motional Training: a pilot study on a novel online training program on social cognition for patients with schizophrenia". *Schizophrenia Research: Cognition*. (4) 10-17.

Wölwer, W., Fromman, N., Halfmann, S., Piaszek, A., Streit, M., y Gaebel, W. 2005. "Remediation of impairments in facial affect recognition in schizophrenia: Efficacy and specificity of a new training program." *Schizophrenia Research* (80), 295-303.
