# Supplementary material for: Improving emotion recognition in schizophrenia with “VOICES”: An on-line prosodic self-training
Source: PLoS One. 2019 Jan 25;14(1):e0210816. doi: 10.1371/journal.pone.0210816 (PMC6347191; doi:10.1371/journal.pone.0210816)
Supplement: S1 Dataset — (DOCX) [file pone.0210816.s004.docx]

**Analysis data *Voices* clinical trial**

**Descriptive analysis:**

|  | Mean | Median | Standard Deviation | Minimum | Maximum |
| --- | --- | --- | --- | --- | --- |
| Age | 40,93 | 45,77 | 12,06 | 20,80 | 69,18 |
| CPZ Equivalence | 1008,30 | 830,00 | 652,75 | 25,0 | 3050,0 |

| **Recruiting Center** | | Frecuency | Percentage | Valid percent | Cumulative percentages |
| --- | --- | --- | --- | --- | --- |
|  | Ourense | 24 | 48,0 | 48,0 | 48,0 |
|  | Ferrol | 8 | 16,0 | 16,0 | 64,0 |
|  | Vigo | 5 | 10,0 | 10,0 | 74,0 |
|  | Otros | 13 | 26,0 | 26,0 | 100,0 |
|  | Total | 50 | 100,0 | 100,0 |  |

| **Sex** | | Frecuency | Percentage | Valid percent | Cumulative percentages |
| --- | --- | --- | --- | --- | --- |
|  | Men | 26 | 52,0 | 52,0 | 52,0 |
|  | Women | 24 | 48,0 | 48,0 | 100,0 |
|  | Total | 50 | 100,0 | 100,0 |  |

|  | | | | | |
| --- | --- | --- | --- | --- | --- |
| **Ocupation** | | Frecuency | Percentage | Valid percent | Cumulative percentages |
|  | Active | 3 | 6,0 | 6,0 | 6,0 |
|  | Inactive | 44 | 88,0 | 88,0 | 94,0 |
|  | Students | 2 | 4,0 | 4,0 | 98,0 |
|  | Marginal Activities | 1 | 2,0 | 2,0 | 100,0 |
|  | Total | 50 | 100,0 | 100,0 |  |

|  | | | | | |
| --- | --- | --- | --- | --- | --- |
| **Studies** | | Frecuencia | Porcentaje | Valid percent | Cumulative percentage |
|  | Without primary studies | 4 | 8,0 | 8,0 | 8,0 |
|  | Primary studies | 11 | 22,0 | 22,0 | 30,0 |
|  | Secondary studies | 27 | 62,0 | 62,0 | 92,0 |
|  | University studies | 1 | 4,0 | 4,0 | 96,0 |
|  | Unknown | 2 | 4,0 | 4,0 | 100,0 |
|  | Total | 50 | 100,0 | 100,0 |  |

|  | | | | | |
| --- | --- | --- | --- | --- | --- |
| **Marital status** | | Frecuencia | Porcentaje | Valid percent | Cumulative percentage |
|  | Single | 42 | 84,0 | 84,0 | 84,0 |
|  | Married | 3 | 6,0 | 6,0 | 90,0 |
|  | Widowed | 1 | 2,0 | 2,0 | 92,0 |
|  | Separated | 4 | 8,0 | 8,0 | 100,0 |
|  | Total | 50 | 100,0 | 100,0 |  |

|  | | | | | |
| --- | --- | --- | --- | --- | --- |
| **Familiar coexistence** | | Frecuencia | Porcentaje | Valid percent | Cumulative percentage |
|  | Alone | 4 | 8,0 | 8,0 | 8,0 |
|  | With parents | 27 | 54,0 | 54,0 | 62,0 |
|  | As a couple | 4 | 8,0 | 8,0 | 70,0 |
|  | Siblings | 1 | 2,0 | 2,0 | 72,0 |
|  | Others | 14 | 28,0 | 28,0 | 100,0 |
|  | Total | 50 | 100,0 | 100,0 |  |

| **Diagnosis** | | Frecuencia | Porcentaje | Valid percent | Cumulative percentage |
| --- | --- | --- | --- | --- | --- |
|  | Schizophrenia | 45 | 90,0 | 90,0 | 90,0 |
|  | Schizoaffective disorder | 5 | 10,0 | 10,0 | 100,0 |
|  | Total | 50 | 100,0 | 100,0 |  |

| **Associated diagnosis** | | Frecuency | Percentage | Valid percent | Cumulative percentage |
| --- | --- | --- | --- | --- | --- |
|  | No other diagnosis | 38 | 76,0 | 76,0 | 76,0 |
|  | Substance-related and addictive disroder (in the past) | 5 | 10,0 | 10,0 | 86,0 |
|  | Mood disorder | 1 | 2,0 | 2,0 | 88,0 |
|  | Somatic sympton and related disorder | 1 | 2,0 | 2,0 | 90,0 |
|  | Personality disorder | 1 | 2,0 | 2,0 | 92,0 |
|  | Neurodevelopmental disorders | 4 | 8,0 | 8,0 | 100,0 |
|  | Total | 50 | 100,0 | 100,0 |  |

| **PSYCOPHARMACOLOGY** | | | | | |
| --- | --- | --- | --- | --- | --- |
| **Benzodiacepines** | | Frecuency | Percentage | Valid percent | Cumulative percentage |
|  | No | 28 | 56,0 | 56,0 | 56,0 |
|  | Yes | 22 | 44,0 | 44,0 | 100,0 |
|  | Total | 50 | 100,0 | 100,0 |  |

|  | | | | | |
| --- | --- | --- | --- | --- | --- |
| **Antidepressants** | | Frecuency | Percentage | Valid percent | Cumulative percentage |
|  | No | 24 | 48,0 | 48,0 | 48,0 |
|  | Yes | 26 | 52,0 | 52,0 | 100,0 |
|  | Total | 50 | 100,0 | 100,0 |  |

|  | | | | | |
| --- | --- | --- | --- | --- | --- |
| **Mood stabilizers** | | Frecuency | Percentage | Valid percent | Cumulative percentage |
|  | No | 41 | 82,0 | 82,0 | 82,0 |
|  | Yes | 9 | 18,0 | 18,0 | 100,0 |
|  | Total | 50 | 100,0 | 100,0 |  |

| 1. **Subgroup analysis** | | | | | |
| --- | --- | --- | --- | --- | --- |
| **Subgroups** | | Frecuency | Percentage | Valid percent | Cumulative percentage |
|  | Control | 24 | 48,0 | 48,0 | 48,0 |
|  | Intervention | 26 | 52,0 | 52,0 | 100,0 |
|  | Total | 50 | 100,0 | 100,0 |  |

|  | | | | | |
| --- | --- | --- | --- | --- | --- |
| **Evaluation tools** | Mean | Median | Standard  Deviation | Minimum | Maximum |
| PANSS | 54,46 | 52,00 | 18,70 | 30 | 100 |
| RMV-SP (pre) | 20,04 | 20,00 | 4,11 | 11,0 | 28,0 |
| RMV-SP (post) | 21,12 | 22,00 | 4,99 | 6,0 | 29,0 |
| K-BIT | 97,90 | 89,00 | 32,20 | 58,0 | 201,0 |

|  | | | | | | |
| --- | --- | --- | --- | --- | --- | --- |
| **Usability test** | Losts | Mean | Medianan | Standard Deviation | Minimum | Maximum |
| FREQ PC | 25 | 2,52 | 3,00 | 1,48 | 0 | 5 |
| FREQ INTERNET | 25 | 2,28 | 2,00 | 1,59 | 0 | 5 |
| EASY CONECTION | 26 | 3,96 | 4,00 | 1,04 | 2 | 5 |
| UNDERSTANDING | 25 | 4,20 | 5,00 | 1,29 | 1 | 5 |
| AUTONOMY | 26 | 3,79 | 4,00 | 1,10 | 1 | 5 |
| ENTERTAINMENT | 25 | 4,04 | 4,00 | 1,06 | 1 | 5 |
| UTILITY | 25 | 3,96 | 4,00 | 1,02 | 2 | 5 |
| IMPROVEMENT | 25 | 3,28 | 4,00 | 1,02 | 1 | 5 |
| NEW RELATIONS | 25 | 3,04 | 3,00 | 1,10 | 1 | 5 |
| IMPROVE WORK | 25 | 3,32 | 4,00 | 1,11 | 1 | 5 |
| DURATION | 25 | 2,72 | 2,00 | 1,28 | 1 | 5 |
| SELF-ESTEEM | 25 | 3,68 | 4,00 | ,90 | 1 | 5 |

|  | | | | | |
| --- | --- | --- | --- | --- | --- |
| **Frequency PC** | | Frequency | Percentage | Valid percent | Cumulative percentage |
| Valid | 0 | 4 | 8,0 | 16,0 | 16,0 |
|  | 1 | 2 | 4,0 | 8,0 | 24,0 |
|  | 2 | 4 | 8,0 | 16,0 | 40,0 |
|  | 3 | 8 | 16,0 | 32,0 | 72,0 |
|  | 4 | 6 | 12,0 | 24,0 | 96,0 |
|  | 5 | 1 | 2,0 | 4,0 | 100,0 |
|  | Total | 25 | 50,0 | 100,0 |  |
| Losts |  | 25 | 50,0 |  |  |
| Total | | 50 | 100,0 |  |  |

|  | | | | | |
| --- | --- | --- | --- | --- | --- |
| **Frequency internet** | | Frequency | Percentage | Valid percent | Cumulative percentage |
| Valid | 0 | 5 | 10,0 | 20,0 | 20,0 |
|  | 1 | 3 | 6,0 | 12,0 | 32,0 |
|  | 2 | 5 | 10,0 | 20,0 | 52,0 |
|  | 3 | 6 | 12,0 | 24,0 | 76,0 |
|  | 4 | 4 | 8,0 | 16,0 | 92,0 |
|  | 5 | 2 | 4,0 | 8,0 | 100,0 |
|  | Total | 25 | 50,0 | 100,0 |  |
| Losts |  | 25 | 50,0 |  |  |
| Total | | 50 | 100,0 |  |  |

|  | | | | | |
| --- | --- | --- | --- | --- | --- |
| **Easy conection** | | Frequency | Percentage | Valid percent | Cumulative percentage |
| Valid | 2 | 4 | 8,0 | 16,7 | 16,7 |
|  | 3 | 1 | 2,0 | 4,2 | 20,8 |
|  | 4 | 11 | 22,0 | 45,8 | 66,7 |
|  | 5 | 8 | 16,0 | 33,3 | 100,0 |
|  | Total | 24 | 48,0 | 100,0 |  |
| Losts |  | 26 | 52,0 |  |  |
| Total | | 50 | 100,0 |  |  |

|  | | | | | |
| --- | --- | --- | --- | --- | --- |
| **Understanding** | | Frequency | Percentage | Valid percent | Cumulative percentage |
| Valid | 1 | 2 | 4,0 | 8,0 | 8,0 |
|  | 2 | 2 | 4,0 | 8,0 | 16,0 |
|  | 4 | 6 | 12,0 | 24,0 | 40,0 |
|  | 5 | 15 | 30,0 | 60,0 | 100,0 |
|  | Total | 25 | 50,0 | 100,0 |  |
| Losts |  | 25 | 50,0 |  |  |
| Total | | 50 | 100,0 |  |  |

|  | | | | | |
| --- | --- | --- | --- | --- | --- |
| **Autonomy** | | Frequency | Percentage | Valid percent | Cumulative percentage |
| Valid | 1 | 1 | 2,0 | 4,2 | 4,2 |
|  | 2 | 3 | 6,0 | 12,5 | 16,7 |
|  | 3 | 2 | 4,0 | 8,3 | 25,0 |
|  | 4 | 12 | 24,0 | 50,0 | 75,0 |
|  | 5 | 6 | 12,0 | 25,0 | 100,0 |
|  | Total | 24 | 48,0 | 100,0 |  |
| Losts |  | 26 | 52,0 |  |  |
| Total | | 50 | 100,0 |  |  |

|  | | | | | |
| --- | --- | --- | --- | --- | --- |
| **Entertaining** | | Frequency | Percentage | Valid percent | Cumulative percentage |
| Valid | 1 | 1 | 2,0 | 4,0 | 4,0 |
|  | 2 | 1 | 2,0 | 4,0 | 8,0 |
|  | 3 | 4 | 8,0 | 16,0 | 24,0 |
|  | 4 | 9 | 18,0 | 36,0 | 60,0 |
|  | 5 | 10 | 20,0 | 40,0 | 100,0 |
|  | Total | 25 | 50,0 | 100,0 |  |
| Losts |  | 25 | 50,0 |  |  |
| Total | | 50 | 100,0 |  |  |

|  | | | | | |
| --- | --- | --- | --- | --- | --- |
| **Utility** | | Frequency | Percentage | Valid percent | Cumulative percentage |
| Valid | 2 | 4 | 8,0 | 16,0 | 16,0 |
|  | 3 | 1 | 2,0 | 4,0 | 20,0 |
|  | 4 | 12 | 24,0 | 48,0 | 68,0 |
|  | 5 | 8 | 16,0 | 32,0 | 100,0 |
|  | Total | 25 | 50,0 | 100,0 |  |
| Losts |  | 25 | 50,0 |  |  |
| Total | | 50 | 100,0 |  |  |

|  | | | | | | | | | | |  |
| --- | --- | --- | --- | --- | --- | --- | --- | --- | --- | --- | --- |
| **Improvement** | | | Frequency | | Percentage | | Valid percent | | Cumulative percentage | |  |
| Valid | 1 | | 1 | | 2,0 | | 4,0 | | 4,0 | |  |
|  | 2 | | 6 | | 12,0 | | 24,0 | | 28,0 | |  |
|  | 3 | | 4 | | 8,0 | | 16,0 | | 44,0 | |  |
|  | 4 | | 13 | | 26,0 | | 52,0 | | 96,0 | |  |
|  | 5 | | 1 | | 2,0 | | 4,0 | | 100,0 | |  |
|  | Total | | 25 | | 50,0 | | 100,0 | |  | |  |
| Losts |  | | 25 | | 50,0 | |  | |  | |  |
| Total | | | 50 | | 100,0 | |  | |  | |  |
|  | | |  | |  | |  | |  | |  |
| **New relations** | | | | Frequency | | Percentage | | Valid percent | | Cumulative percentage | |
| Valid | | 1 | | 3 | | 6,0 | | 12,0 | | 12,0 | |
|  |  | 2 | | 4 | | 8,0 | | 16,0 | | 28,0 | |
|  |  | 3 | | 8 | | 16,0 | | 32,0 | | 60,0 | |
|  |  | 4 | | 9 | | 18,0 | | 36,0 | | 96,0 | |
|  |  | 5 | | 1 | | 2,0 | | 4,0 | | 100,0 | |
|  |  | Total | | 25 | | 50,0 | | 100,0 | |  | |
| Losts | |  | | 25 | | 50,0 | |  | |  | |
| Total | | | | 50 | | 100,0 | |  | |  | |

|  | | | | | |
| --- | --- | --- | --- | --- | --- |
| **Improvement at work** | | Frequency | Percentage | Valid percent | Cumulative percentage |
| Valid | 1 | 2 | 4,0 | 8,0 | 8,0 |
|  | 2 | 4 | 8,0 | 16,0 | 24,0 |
|  | 3 | 5 | 10,0 | 20,0 | 44,0 |
|  | 4 | 12 | 24,0 | 48,0 | 92,0 |
|  | 5 | 2 | 4,0 | 8,0 | 100,0 |
|  | Total | 25 | 50,0 | 100,0 |  |
| Losts |  | 25 | 50,0 |  |  |
| Total | | 50 | 100,0 |  |  |

|  | | | | | |
| --- | --- | --- | --- | --- | --- |
| **Duration** | | Frequency | Percentage | Valid percent | Cumulative percentage |
| Valid | 1 | 4 | 8,0 | 16,0 | 16,0 |
|  | 2 | 10 | 20,0 | 40,0 | 56,0 |
|  | 3 | 2 | 4,0 | 8,0 | 64,0 |
|  | 4 | 7 | 14,0 | 28,0 | 92,0 |
|  | 5 | 2 | 4,0 | 8,0 | 100,0 |
|  | Total | 25 | 50,0 | 100,0 |  |
| Losts |  | 25 | 50,0 |  |  |
| Total | | 50 | 100,0 |  |  |

|  | | | | | |
| --- | --- | --- | --- | --- | --- |
| **Self-esteem** | | Frequency | Percentage | Valid percent | Cumulative percentage |
| Valid | 1 | 1 | 2,0 | 4,0 | 4,0 |
|  | 2 | 1 | 2,0 | 4,0 | 8,0 |
|  | 3 | 6 | 12,0 | 24,0 | 32,0 |
|  | 4 | 14 | 28,0 | 56,0 | 88,0 |
|  | 5 | 3 | 6,0 | 12,0 | 100,0 |
|  | Total | 25 | 50,0 | 100,0 |  |
| Losts |  | 25 | 50,0 |  |  |
| Total | | 50 | 100,0 |  |  |

**Chi square test:**

| **Contingency table** | | | | | |
| --- | --- | --- | --- | --- | --- |
|  | | | GROUP | | Total |
|  |  |  | Control | Intervention |  |
| SEX | Men | Count | 13 | 13 | 26 |
|  |  | % in GROUP | 54,2% | 50,0% | 52,0% |
|  | Women | Count | 11 | 13 | 24 |
|  |  | % in GROUP | 45,8% | 50,0% | 48,0% |
| Total | | Count | 24 | 26 | 50 |
|  |  | % in GROUP | 100,0% | 100,0% | 100,0% |

P=0,768

| **Contingency table** | | | | | |
| --- | --- | --- | --- | --- | --- |
|  | | | GROUP | | Total |
|  |  |  | Control | Intervention |  |
| OCUPATION | Active | Count | 2 | 1 | 3 |
|  |  | % in GROUP | 8,3% | 3,8% | 6,0% |
|  | Inactive | Count | 21 | 23 | 44 |
|  |  | % in GROUP | 87,5% | 88,5% | 88,0% |
|  | Student | Count | 0 | 2 | 2 |
|  |  | % in GROUP | 0,0% | 7,7% | 4,0% |
|  | Marginal activity | Count | 1 | 0 | 1 |
|  |  | % in GROUP | 4,2% | 0,0% | 2,0% |
| Total | | Count | 24 | 26 | 50 |
|  |  | % in GROUP | 100,0% | 100,0% | 100,0% |
|  | |  |  |  |  |

P=0,362 chi

|  | | | | | |
| --- | --- | --- | --- | --- | --- |
|  | | | GROUP | | Total |
|  |  |  |  | |  |
|  |  |  | Control | Intervention |  |
| Marital status | Single | Count | 19 | 23 | 42 |
|  |  | % in GROUP | 79,2% | 88,5% | 84,0% |
|  | Married | Count | 1 | 2 | 3 |
|  |  | % in GROUP | 4,2% | 7,7% | 6,0% |
|  | Widowed | Count | 1 | 0 | 1 |
|  |  | % in GROUP | 4,2% | 0,0% | 2,0% |
|  | Separated | Count | 3 | 1 | 4 |
|  |  | % in GROUP | 12,5% | 3,8% | 8,0% |
| Total | | Count | 24 | 26 | 50 |
|  |  | % in GROUP | 100,0% | 100,0% | 100,0% |

| **Contingency table** | | | | | |
| --- | --- | --- | --- | --- | --- |
|  | | | GROUP | | Total |
|  |  |  | Control | Intervention |  |
| Educational level | Without primary studies | Count | 3 | 1 | 4 |
|  |  | % in GROUP | 12,5% | 3,8% | 8,0% |
|  | Primary studies | Count | 3 | 8 | 11 |
|  |  | % in GROUP | 12,5% | 30,8% | 22,0% |
|  | Secondary studies | Count | 15 | 15 | 30 |
|  |  | % in GROUP | 62,5% | 61,6% | 62,0% |
|  | Universitary studies | Count | 2 | 0 | 2 |
|  |  | % in GROUP | 8,3% | 0,0% | 4,0% |
|  | Unknown | Count | 1 | 1 | 2 |
|  |  | % in GROUP | 4,2% | 3,8% | 4,0% |
| Total | | Count | 24 | 26 | 50 |
|  |  | % in GROUP | 100,0% | 100,0% | 100,0% |
| P=0.362 | |  |  |  |  |
|  | | **Contingency** | table |  |  |

P=0,451

| **Contingency table** | | | | | |
| --- | --- | --- | --- | --- | --- |
|  | | | GROUP | | Total |
|  |  |  | Control | Intervention |  |
| Familiar coexistence | Alone | Count | 2 | 2 | 4 |
|  |  | % in GROUP | 8,3% | 7,7% | 8,0% |
|  | With parents | Count | 14 | 13 | 27 |
|  |  | % in GROUP | 58,3% | 50,0% | 54,0% |
|  | As a couple | Count | 2 | 2 | 4 |
|  |  | % in GROUP | 8,3% | 7,7% | 8,0% |
|  | Siblings | Count | 1 | 0 | 1 |
|  |  | % in GROUP | 4,2% | 0,0% | 2,0% |
|  | Others | Count | 5 | 9 | 14 |
|  |  | % in GROUP | 20,8% | 34,6% | 28,0% |
| Total | | Count | 24 | 26 | 50 |
|  |  | % in GROUP | 100,0% | 100,0% | 100,0% |

P=0,717chi

| **Fisher test:**  **Contingency table** | | | | | |
| --- | --- | --- | --- | --- | --- |
|  | | | GROUP | | Total |
|  |  |  | Control | Intervention |  |
| Diagnose | Schizophrenia | Count | 21 | 24 | 45 |
|  |  | % in GROUP | 87,5% | 92,3% | 90,0% |
|  | Schizoaffective disorder | Count | 3 | 2 | 5 |
|  |  | % in GROUP | 12,5% | 7,7% | 10,0% |
| Total | | Count | 24 | 26 | 50 |
|  |  | % in GROUP | 100,0% | 100,0% | 100,0% |

P=0,461

| **Contingency table** | | | | | |
| --- | --- | --- | --- | --- | --- |
|  | | | GROUP | | Total |
|  |  |  | Control | Intervention |  |
| BZD | No | Count | 13 | 15 | 28 |
|  |  | % in GROUP | 54,2% | 57,7% | 56,0% |
|  | Yes | Count | 11 | 11 | 22 |
|  |  | % in GROUP | 45,8% | 42,3% | 44,0% |
| Total | | Count | 24 | 26 | 50 |
|  |  | % in GROUP | 100,0% | 100,0% | 100,0% |
| P=0,802 | |  |  |  |  |

| **Contingency table** | | | | | |
| --- | --- | --- | --- | --- | --- |
|  | | | GROUP | | Total |
|  |  |  | Control | Intervention |  |
| Antidepressant | No | Count | 13 | 11 | 24 |
|  |  | % in GROUP | 54,2% | 42,3% | 48,0% |
|  | Si | Count | 11 | 15 | 26 |
|  |  | % in GROUP | 45,8% | 57,7% | 52,0% |
| Total | | Count | 24 | 26 | 50 |
|  |  | % in GROUP | 100,0% | 100,0% | 100,0% |

P=0.402 chi

| **Contingency table** | | | | | |
| --- | --- | --- | --- | --- | --- |
|  | | | GROUP | | Total |
|  |  |  | Control | Intervention |  |
| Eutimizante | No | Count | 18 | 23 | 41 |
|  |  | % in GROUP | 75,0% | 88,5% | 82,0% |
|  | Yes | Count | 6 | 3 | 9 |
|  |  | % in GROUP | 25,0% | 11,5% | 18,0% |
| Total | | Count | 24 | 26 | 50 |
|  |  | % in GROUP | 100,0% | 100,0% | 100,0% |

P=0.193 Fisher´s exact test

**Kolmogorov-Smirnov test for a sample**

|  | | | | | |
| --- | --- | --- | --- | --- | --- |
|  | RMV-SP (i) | Voices S3 | Voices S7 | RMV-SP (f) | FREQ |
| Sig. asintótica (bilateral) | ,089 | ,200^d^ | ,105 | ,097 | ,128 |

|  | | | |
| --- | --- | --- | --- |
| GROUP | | RMV-SV (i) | RMV-SV (f) |
| Control | Mean | 20,08 | 19,25 |
|  | Standard deviation | 4,23 | 4,93 |
|  | Median | 20,50 | 18,50 |
|  | Minimum | 11,0 | 6,0 |
|  | Maximum | 28,0 | 29,0 |
| Intervention | Mean | 20,00 | 22,92 |
|  | Standard deviation | 4,08 | 4,43 |
|  | Median | 20,00 | 24,00 |
|  | Minimum | 12,0 | 11,0 |
|  | Maximum | 28,0 | 29,0 |
| p-value |  | 0.944 | 0.009 |

**Mann-Whitney non-parametric test:**

| GROUP | | Age | CPZ equivalence | PANSS | K-BIT |
| --- | --- | --- | --- | --- | --- |
| Control | Mean | 41,17 | 1102,08 | 56,75 | 96,92 |
|  | Standard deviation | 12,14 | 725,27 | 19,32 | 33,52 |
|  | Median | 45,95 | 1180,00 | 55,00 | 84,50 |
|  | Minimum | 23,65 | 25,0 | 30 | 63,0 |
|  | Maximum | 69,18 | 3050,0 | 100 | 201,0 |
| Intervention | Mean | 40,71 | 921,73 | 52,35 | 98,81 |
|  | Standard deviation | 12,22 | 578,74 | 18,23 | 31,56 |
|  | Median | 44,95 | 775,00 | 44,00 | 90,50 |
|  | Minimum | 20,80 | 80,0 | 30 | 58,0 |
|  | Maximum | 63,03 | 2412,0 | 91 | 180,0 |
| p-value |  | ,969 | ,398 | ,346 | ,478 |

|  | GROUP Control | | | | | p-value |
| --- | --- | --- | --- | --- | --- | --- |
|  | Mean | Standard deviation | Median | Minimum | Maximum |  |
| RMV-SV (i) | 20,083 | 4,2315 | 20,500 | 11,0 | 28,0 | 0.153 |
| RMV-SV (f) | 19,250 | 4,9277 | 18,500 | 6,0 | 29,0 |  |

**Paired Student´s T test:**

There are no significant differences in pre-post in control group.

|  | GROUP Intervention | | | | | p-value |
| --- | --- | --- | --- | --- | --- | --- |
|  | Mean | Standard  Deviation | Median | Minimum | Maximum |  |
| RMV-SV (i) | 20,000 | 4,0792 | 20,000 | 12,0 | 28,0 | <0.001 |
| RMV-SV (f) | 22,920 | 4,4340 | 24,000 | 11,0 | 29,0 |  |

There are no significant differences in pre-post in intervention group.
