# Supplementary material for: Improving emotion recognition in schizophrenia with “VOICES”: An on-line prosodic self-training
Source: PLoS One. 2019 Jan 25;14(1):e0210816. doi: 10.1371/journal.pone.0210816 (PMC6347191; doi:10.1371/journal.pone.0210816)
Supplement: S2 Dataset — (DOCX) [file pone.0210816.s005.docx]

**ANOVA TEST**

| **Cases description** | | | | | |
| --- | --- | --- | --- | --- | --- |
|  | | | | | |
| GROUP | Mean | Standard  Deviation | Median | Minimum | Maximum |
| Control | -,8333 | 2,76101 | ,0000 | -7,00 | 4,00 |
| Intervention | 2,7600 | 3,55059 | 3,0000 | -5,00 | 11,00 |
| Total | 1,0000 | 3,64005 | 1,0000 | -7,00 | 11,00 |

| **Test of Between-Subjects Effects** | | | | | | | |
| --- | --- | --- | --- | --- | --- | --- | --- |
| Dependent Variable: DIF RMVS | | | | | | | |
| Source | | Type III Sum of squares | df | Mean Square | F | Sig. | Partial Eta Squared |
| K-BIT |  | 3,915 | 1 | 3,915 | ,380 | ,541 | ,008 |
| GROUP |  | 171,642 | 2 | 85,821 | 8,329 | ,001 | ,266 |
